# EP2 and EP4 blockade prevents tumor-induced suppressive features in human monocytic myeloid-derived suppressor cells

**DOI:** 10.3389/fimmu.2024.1355769

**Published:** 2024-01-26

**Authors:** Jorge Cuenca-Escalona, Beatriz Subtil, Alba Garcia-Perez, Alessandra Cambi, I. Jolanda M. de Vries, Georgina Flórez-Grau

**Affiliations:** Department of Medical BioSciences, Radboud University Medical Center, Nijmegen, Netherlands

**Keywords:** M-MDSCs, prostaglandin E2, E-prostanoid receptor type 2, E-prostanoid receptor type 4, tumor microenvironment

## Abstract

Tumors educate their environment to prime the occurrence of suppressive cell subsets, which enable tumor evasion and favors tumor progression. Among these, there are the myeloid-derived suppressor cells (MDSCs), their presence being associated with the poor clinical outcome of cancer patients. Tumor-derived prostaglandin E2 (PGE2) is known to mediate MDSC differentiation and the acquisition of pro-tumor features. In myeloid cells, PGE2 signaling is mediated via E-prostanoid receptor type 2 (EP2) and EP4. Although the suppressive role of PGE2 is well established in MDSCs, the role of EP2/4 on human MDSCs or whether EP2/4 modulation can prevent MDSCs suppressive features upon exposure to tumor-derived PGE2 is poorly defined. In this study, using an *in vitro* model of human monocytic-MDSCs (M-MDSCs) we demonstrate that EP2 and EP4 signaling contribute to the induction of a pro-tumor phenotype and function on M-MDSCs. PGE2 signaling via EP2 and EP4 boosted M-MDSC ability to suppress T and NK cell responses. Combined EP2/4 blockade on M-MDSCs during PGE2 exposure prevented the occurrence of these suppressive features. Additionally, EP2/4 blockade attenuated the suppressive phenotype of M-MDSCs in a 3D coculture with colorectal cancer patient-derived organoids. Together, these results identify the role of tumor-derived PGE2 signaling via EP2 and EP4 in this human M-MDSC model, supporting the therapeutic value of targeting PGE2-EP2/4 axis in M-MDSCs to alleviate immunosuppression and facilitate the development of anti-tumor immunity.

## Introduction

Tumors initiate a variety of suppressive mechanisms that impair the development of anti-tumor immunity. To this end, tumor cells shape their environment to establish the so-called suppressive tumor microenvironment (TME). The TME is composed by tumor, stromal, immune cells, and soluble factors that enable the occurrence of regulatory networks across the host immune system (1–4). Among these TME-educated cells, the myeloid-derived suppressor cells (MDSCs), described in a variety of human cancers (5–12) are associated with a poor patient prognosis (5, 6, 13). MDSCs are a heterogeneous group of immature myeloid cells phenotypically distinguished by the expression of CD11b^+^ CD33^+^ HLA-DR^-/low^ and can be further subdivided into two subgroups: monocytic MDSCs (M-MDSCs) and polymorphonuclear MDSCs (PMN-MDSCs) characterized by the expression of CD14 and CD15 respectively (14, 15). MDSCs exploit multiple mechanisms to hamper anti-tumor immunity such as the expression of transforming growth factor-beta, reactive oxygen species, prostaglandin E2 (PGE2), IL-10, arginase-1, and indoleamine dioxygenase (16–22). Functionally, MDSCs hamper anti-tumor immunity by impairing T cell proliferation, IFNγ responses, and NK cell function (20, 21, 23–29) while facilitating the expansion of regulatory T cells (Tregs) (9, 30, 31), thus contributing to tumor evasion.

To date, different TME-derived soluble factors have been identified as modulators of MDSCs, including GM-CSF, IL-6, VEGF, and PGE2 (20, 24, 32, 33). In particular, studies with human cells have demonstrated the crucial role of tumor-derived PGE2 during the development of fully functional MDSCs (20, 24, 28, 34, 35). PGE2 is a lipid mediator synthesized via the activity of the enzyme cyclooxygenase-1/2 (COX-1/2) (36). Noteworthy, studies in mice have demonstrated that PGE2 ablation impairs the occurrence of suppressive MDSCs, enabling anti-tumor immunity and hampering tumor growth (28, 34, 35). Besides MDSCs, tumor-derived PGE2 is well-known to prime tolerogenic functions across various immune cell compartments (37–39), underscoring PGE2 signaling as an interesting target for cancer treatment. Although the pharmacological intervention of COX1/2 has demonstrated to protect against cancer prevalence and progression (40–43), the occurrence of severe side effects has impeded its establishment as an anti-tumor therapy (44, 45), arguing for the need to investigate alternative strategies to target PGE2 signaling in the TME. PGE2 primes immunosuppression in the immune system through the E-prostanoid receptor type 2 (EP2) and EP4 (39, 46–48). In MDSCs, PGE2-EP2/4 signaling is involved in the development of suppressive features (20, 28, 49–53). EP2/4-mediated effects on MDSCs are IL-10 production (20, 53), COX-2 upregulation (20), or the ability to suppress anti-tumor NK (28) and T cell responses (20, 51, 53, 54). Noteworthy, EP2/4 targeting, or ablation attenuates the development of suppressive MDSCs and prevents tumor growth in mice (49, 50, 52, 53). Although targeting the PGE2-EP2/4 axis has proven of therapeutic value in mice models, the exact role of PGE2-EP2/4 as well as the relevance of targeting this axis in human MDSCs is still poorly defined, likely due to the scarcity of these cells in human blood that hampers in-depth mechanistic studies. Given the key role of PGE2 as MDSC modulator, it is essential to further investigate the function of EP2 and EP4 receptors and signaling in human MDSCs. Prior studies investigating the role of PGE2-EP2/4 axis in cells of the myeloid lineage used human monocytes or monocyte-derived DCs (moDCs) (25, 28, 50, 53). We here used *in vitro* generated human monocyte-derived MDSCs (moMDSCs), a cell model recently reported to recapitulate the phenotype and function of human M-MDSCs from cancer patients (21, 31, 55).

In this study, we examine the role of EP2 and EP4 signaling in the differentiation, phenotype, and function of moMDSCs upon tumor-derived PGE2 contact. We show that exposure of human monocytes to tumor-derived PGE2 leads to a suppressive state that resembles that of M-MDSCs and is attenuated upon EP/EP4 blockade. Using a human M-MDSC model, we show that tumor-derived PGE2 steers moMDSCs towards the acquisition of a suppressive phenotype as seen by the upregulation of CD14, IL-10 and PGE2 production, or the downregulation of HLA-DR. Additionally, PGE2-EP2/4 signaling boosts the suppressive function of moMDSCs over the development of pro-inflammatory responses in NK cells, T cells, and moDCs. Blockade of EP2 and EP4 demonstrated that both receptors contribute to the acquisition of this suppressive state, with EP4 rather than EP2 mediating this effect. Combined EP2/4 blockade completely impairs the arising of such phenotype and function. Using a 3D co-culture with M-MDSCs and colorectal cancer patient-derived organoids, blockade of EP2 and EP4 attenuates the suppressive phenotype of moMDSCs. Altogether, these results illustrate the role of EP2 and EP4 in human M-MDSCs as well as the relevance of targeting the PGE2-EP2/4 axis in human MDSCs for the treatment of cancer.

## Materials and methods

### Cell isolation

Monocytes, pan T cells, naïve CD4 T cells, and NK cells were obtained from peripheral blood mononuclear cells (PBMCs), isolated by Ficoll density gradient centrifugation (Lymphoprep, Serumwerk Bernburg) from buffy coats derived from healthy blood donors (Sanquin, the Netherlands). Monocytes were isolated with MACS CD14+ isolation kit (130-050-201, Miltenyi Biotec). After the positive CD14 isolation, the negative fraction containing the peripheral blood lymphocytes (PBLs) was used to isolate autologous pan T cells and NK cells. Pan T cells and NK cells were isolated using the MACS Pan T cell isolation kit (130-096-535, Miltenyi Biotec) and the NK cell isolation kit (130-092-657, Miltenyi Biotec) following manufacturer’s instructions. Naïve CD4 T cells were isolated from PBLs using the MACS Naïve CD4 T cell isolation kit (130-094-131, Miltenyi Biotec) following the manufacturer’s instructions. Pan T cells, NK cells, and naïve CD4 T cells were cryopreserved until use in freezing media, composed of 50% X-VIVO medium (Lonza), 40% FBS (HyClone) and 10% DMSO (WAK-Chemie).

### *In vitro* generation of moMDSCs and moDCs

moMDSCs and moDCs were differentiated *in vitro* by culturing monocytes at a concentration of 0.5 million cells per ml in T25 flasks containing a final volume of 4 ml X-VIVO medium (Lonza) supplemented with 2% human serum (HS) (Sanquin, the Netherlands) for 7 days. For the generation of moMDSCs, monocyte cultures were supplemented with 60 U/ml of GM-CSF (130-093-867, Miltenyi Biotec) and 10 ng/ml of IL-6 (130-093-933, Miltenyi Biotec). For moDC generation, monocyte cultures were supplemented with 450 U/ml of GM-CSF (130-093-867, Miltenyi Biotec) and 300 U/ml of IL-4 (130-093-924, Miltenyi Biotec). On day 3 of differentiation, moMDSCs and moDC cultures were supplemented with 1 ml of X-VIVO supplemented with 2% HS and 5x concentrated IL-6 and GM-CSF in the case of moMDSCs, and 5x concentrated IL-4 and GM-CSF in the case of moDCs. To generate mature moDCs, on day 6 of differentiation, moDC cultures were further stimulated with 2 µg/ml of poly (I:C) (Invivogen) for 24 hours prior to further use in functional experiments.

### Cell culture and conditioned media preparation

Human melanoma cells (A375) were tested to be mycoplasma-free and cultured in Dulbecco’s modified Eagle’s medium (DMEM, Gibco) supplemented with 10% fetal bovine serum (FBS) 1% Antibiotic-Antimycin (AA, Gibco). To generate the melanoma cell line conditioned media (CM), A375 cells were cultured in T75 flasks at a concentration of 0.3 million cells per ml in T75 flasks with a final volume of 14 ml. After three days of culture, the supernatant was harvested and centrifuged for 5 minutes at 1500 rpms to get rid of cellular debris present in the media. Resulting CM was aliquoted and stored at -20 C until use.

### MoMDSC stimulation

To determine the individual role of EP2 and EP4 on moMDSCs, the specific EP2 antagonist AH6809 (Cayman chemicals) and specific EP4 antagonist L161-982 (Cayman chemicals) were used. For EP2 blockade, 40 µM of EP2 antagonist was used, whereas 3 µM was used for the blocking of EP4 receptor. The same concentrations were used when performing the combined EP2/4 blockade. On day 5 of moMDSC differentiation, cultures were treated with the indicated antagonist two hours prior to exposure to the melanoma-derived CM. After 3 hours, 2 ml of CM were added to each T25 flask.

To establish the role of CM addition, moMDSCs were instead provided with 2 ml of fresh media, depicted as untreated moMDSCs. On day 7, after 48 hours of CM treatment, moMDSCs were harvested. For harvesting, moMDSCs culture media was removed and replaced by cold PBS supplemented with 0.1% BSA and 0.2 mM of EDTA followed by incubation for 1 hour at 4**°**C. Thereafter, flasks were tapped and moMDSCs cell suspension was harvested and centrifuged prior to further analysis.

### Generation of CM educated monocytes

The impact of CM on monocyte development was tested by culturing monocytes at a concentration of 0.5 million cells per ml in 6 well plates with 2 ml of X-VIVO media supplemented with 2% HS. To determine the role of EP2/4 during differentiation, monocyte cultures were supplemented with 40 µM and 3 µM of EP2 antagonist and EP4 antagonist respectively. After 2 hours, monocyte cultures were treated with 0.5 ml of CM. On day 3, the differentiated monocytes were harvested for flow cytometric analysis. For day 6 of differentiation, day 3 monocytes were re-stimulated with the indicated concentrations of aEP2/4 and exposed to a second dose of 0.5 ml CM. On day 6, the resulting differentiated monocytes were harvested and either analyzed by flow cytometry or replated for the performance of functional experiments.

### Flow cytometric analysis of moMDSCs and differentiated monocytes

Flow cytometry was used to determine the phenotype of moMDSCs and the differentiated monocytes. Cells were harvested from culture flask or plates, washed with cold PBS, and incubated with blocking buffer (PBS + 0.1% BSA + 0.01 NaH3 + 5% HS). After 15 minutes, cells were stained with the specific antibodies for CD14 APC-H7 (1:100, 560180, BD Biosciences), CCR5 BV421 (1:50, 359118, Biolegend), CD86 APC (1:100, 555660, BD Biosciences), CD11b Pe (1:100, 301306, Biolegend), HLA-DR BV510 (1:100, 307646), and MerTK Pe-Cy7 (1:100, 367610, Biolegend). After staining for 20 minutes, cells were washed twice using blocking buffer prior to flow cytometric analysis (see **Supplementary Figures 1B** and **2** for the gating strategy). Flow cytometric analysis was performed on the BD FACS Verse, and data were analyzed in FlowJo software version (BD, Franklin Lakes, NJ, USA).

### Cytokine and PGE2 production measurement

For the detection of IL-6, IL-10, and PGE2 production, harvested moMDSCs were replated in 96 U bottom plates at a concentration of 0.5 million cells per ml at a final volume of 200 µL of X-VIVO medium supplemented with 2% HS and 100 ng/ml LPS. After 24 hours of culture, the moMDSCs supernatants were harvested and analyzed by ELISA for the presence of IL-6 (88-7106-77, Invitrogen), IL-10 (88-7066-77 Invitrogen), and PGE2 (EHPGE2 ThermoFisher) according to manufacturer’s instructions.

### Autologous T cell suppression assays

The suppression of autologous T cell responses by moMDSCs was assessed by coculturing moMDSCs in 96 U bottom plates with autologous T cells. Prior to coculture, T cells were cultured for 30 minutes in X-VIVO supplemented with 2% HS at a concentration of 1 million cells per ml with human activator anti-CD3/CD28 dynabeads (1:100, 11161D, ThermoFisher). For T cell proliferation assays, autologous pan T cells were stained with 5 µM carboxyfluorescein diacetate succinimidyl ester (CFSE, C34554, ThermoFisher). moMDSCs were cultured with CFSE-labelled T- cells in a ratio 1:1 for three days at 37°C, 5% CO2. After three days, T cells were harvested and stained in cold PBS with the viability dye eFluor 780 (1:2000, ThermoFisher) for 15 minutes followed by CD4 PE staining (1:100, 555347 BD Biosciences) and CD8 APC (1:100, 555369, BD Biosciences) for 20 minutes. T cell proliferation was determined as loss of CFSE dye in T cells (see **Supplementary Figure 3A**)

To determine cytokine production by T cells, moMDSCs were cocultured with autologous pan T cells non-CFSE labelled at a ratio 1:1. After three days, T cells were stimulated with 25 ng/ml of PMA (Calbiochem), 0.5 mg/ml of Ionomycin (I0634, Sigma-Aldrich) and 10 ng/ml of brefeldin A (Cayman chemicals). After 3 hours of stimulation, T cells were washed and stained with the viability dye eFluor 780 (1:2000, ThermoFisher) for 15 minutes in cold PBS. Next, cells were fixated and permeabilized by using the FOXP3 kit (00-5523-00, ThermoFisher) according to manufacturer’s instructions. Cells were then blocked in wash and permeabilization buffer (provided by the kit) supplemented with 5% HS. After 15 minutes, T cells were washed and stained for 20 minutes with antibodies for IFNγ BV421 (1:100, 562988, BD Biosciences), IL-2 PerCP-Cy5.5 (1:100, 500322, Biolegend), TNFα APC (1:50, 130-117-382, Miltenyi Biotec), and CD8 BV510 (1:100, 344732, Biolegend). Next, cells were washed twice using blocking buffer prior to flow cytometric analysis (see **Supplementary Figure 3B** for gating strategy). Flow cytometric analysis was performed on the BD FACS Verse, and data were analyzed in FlowJo software version (BD, Franklin Lakes, NJ, USA).

### Autologous NK cell suppression assays

The suppression of autologous NK cell responses by moMDSCs was assessed by coculturing moMDSCs in 96 U bottom culture plates with autologous NK cells. Prior to coculture, NK cells were cultured for 1 hour in X-VIVO supplemented with 2% HS and 100 U/ml of IL-2 (130-097-773, Miltenyi Biotec) at a concentration of 1 million cells per ml. Then moMDSCs were cocultured with NK cells at a ratio 1:2 (M-MDSC: NK cell ratio) for three days at 37°C, 5% CO2. For the characterization of activation markers, NK cells were harvested and stained in cold PBS with the viability dye eFluor 780 (1:2000, ThermoFisher) for 15 minutes. Next, NK cells were incubated with blocking buffer (PBS + 0.1% BSA + 0.01 NaH3 + 5% HS). After 15 minutes, cells were stained with the specific antibodies for NKG2D BV510 (1:50, 320816, Biolegend), NKp46 Pe (1:50, IM3711, Beckman), CD25 APC (1:100, 555434, BD Biosciences), CD69 PerCP (1:50, 340548, Biolegend), and CD56 BV421 (1:100, 562751, BD Biosciences). After staining for 20 minutes, cells were washed twice using blocking buffer prior to flow cytometric analysis (see **Supplementary Figure 4** for gating strategy). Flow cytometric analysis was performed on the BD FACS Lyric, and data were analyzed in FlowJo software version (BD, Franklin Lakes, NJ, USA).

For the characterization of intracellular cytokine levels, NK cells were stimulated with 25 ng/ml of PMA (Calbiochem), 0.5 mg/ml of Ionomycin (Sigma-Aldrich) and 10 ng/ml of brefeldin A (Cayman chemicals) for 3 hours. Next, NK cells were washed and stained with the viability dye eFluor 780 (1:2000, ThermoFisher) for 15 minutes in cold PBS. Then, cells were fixated and permeabilized by using the FOXP3 kit (00-5523-00, ThermoFisher) according to manufacturer’s instructions. Prior to staining, cells were incubated in wash and permeabilization buffer (provided by the kit) supplemented with 5% HS. After 15 minutes, T cells were washed and stained for 20 minutes with antibodies specific for IFNγ BV421 (1:100, 562988, BD Biosciences), granzyme b Pe (1:100, 372208, Biolegend), TNFα PerCP-Cy5.5 (1:100, 45-7349-42, eBioscience), and CD56 APC (1:50, 341027, BD Biosciences). Next, cells were washed twice using blocking buffer prior to flow cytometric analysis (see **Supplementary Figure 4** for the gating strategy). Flow cytometric analysis was performed on the BD FACS Verse, and data were analyzed in FlowJo software version (BD, Franklin Lakes, NJ, USA).

### MoMDSCs suppression of moDC mediated T cell polarization assay

The ability of moMDSCs to modulate the expansion of pro-inflammatory T cell populations by activated DCs was determined by coculturing moDCs, allogeneic naïve CD4 T cells, and moMDSCs at a ratio 1:10:5 in 96 U bottom plates. On day 6 of coculture, half of the culture medium containing the expanding T cells was removed. Equal volume of fresh X-VIVO medium supplemented with 2% HS was added and T cell cultures were further supplemented with 20 U/ml of IL-2 (130-097-773, Miltenyi Biotec). From this day onwards, cell culture media was refreshed as described above with new media containing 20 U/ml of IL-2 every 2 days until day 12 of coculture. Resulting T cell populations were harvested and characterized either for the presence of Tregs or the intracellular cytokine expression. For determining the presence of Tregs, T cells were stained with the viability dye eFluor 780 (1:2000, ThermoFisher) in cold PBS for 15 minutes. T cells were then fixed using the FOXP3 staining kit (00-5523-00, ThermoFisher) according to manufacturer’s instructions. After fixation, cells were incubated in wash/permeabilization buffer supplemented with 5% HS for 15 minutes. Then, cells were washed and stained for CD127 PE (1:100 12-12780-42, eBioScience), CD25 APC (1:100 555434, BD Biosciences), and FOXP3 A488 (1:50 53-4776-42, eBioscience) for 20 minutes. Stained cells were washed twice prior to flow cytometric analysis (see **Supplementary Figure 5B** for gating strategy).

For the characterization of intracellular cytokines, T cells were stimulated with 25 ng/ml of PMA (Calbiochem), 0.5 mg/ml of Ionomycin (Sigma-Aldrich) and 10 ng/ml of brefeldin A (Cayman chemicals) for 3 hours. Next, T cells were washed and stained with the viability dye eFluor 780 (1:2000, ThermoFisher) for 15 minutes in cold PBS. Then, cells were fixated and permeabilized by using the FOXP3 kit (00-5523-00, ThermoFisher) according to manufacturer’s instructions. T cells were blocked in wash and permeabilization buffer (provided by the kit) supplemented with 5% HS. After 15 minutes, T cells were washed and stained for 20 minutes with antibodies specific for IFNγ BV421 (1:100, 562988, BD Biosciences), TNFα Pe (1:100, 502909, Biolegend), or IL-2 PerCP-Cy5.5 (1:100, 500322, eBioscience). Next, cells were washed twice and analyzed by flow cytometry (see **Supplementary Figure 5A** for gating strategy). Flow cytometry was performed on the BD FACS Verse, and data were analyzed in FlowJo software version (BD, Franklin Lakes, NJ, USA).

### 3D coculture of M-MDSCs with CRC-PDTOs

CRC-PDTOs (originally named PDO013 in the biobank (56) were established and cultured as described before (57). The generation of cocultures between PDTOs and moMDSCs in a 3D collagen gel was performed as previously described for DC studies (57). In short, PDTOs and moMDSCs on day 5 of differentiation were embedded in a collagen matrix at a cell ratio of 1:1. Collagen drops (of 25 µL containing 50 000 PDTOs and moMDSCs) were loaded into 24 well plates and incubated at 37**°**C to enable collagen polymerization and solidification for 45 minutes. Collagen drops were cultured with 300 µL X-VIVO media supplemented with 2% HS. Next day, cultures were either treated with or without the EP2/4 antagonists. For this experiment, 120 µM and 9 µM of EP2 and EP4 antagonists respectively were used. After 24 hours, collagen was degraded using collagenase I (Sigma-Aldrich, C0130). Dissociated cell suspension was then filtered, and the resulting cell suspension was washed and stained with antibodies specific for CD11b Pe (1:100, 301306, Biolegend), HLA-DR BV510 (1:100, 307646), MerTK Pe-Cy7 (1:100, 367610, Biolegend), CD14 APC-H7 (1:100, 560180, BD Biosciences), CCR5 BV421 (1:50, 359118, Biolegend), and CD86 APC (1:100, 555660, BD Biosciences). Next, cells were washed and analyzed by flow cytometry using the BD FACS Verse (see **Supplementary Figure 6** for gating strategy), and data were analyzed in FlowJo software version (BD, Franklin Lakes, NJ, USA).

### Statistical analysis

Statistical analysis was performed with Graphpad Prism 8 (Version 8.0.2. GraphPad Software San Diego, CA, USA). Unless indicated otherwise, data are depicted as mean ± SEM in bar scattered dot plots. Significance was determined as indicated in the legend of each figure. MoMDSCs phenotype data were analyzed using either a one-way analysis of variance (ANOVA) followed by multiple comparison Tukey correction, or a Friedman test followed by Dunn’s multiple comparison test. Significance across the functional experiments was determined using either a one-way ANOVA followed by a Dunnett’s test, or a Friedman test followed by a Dunn’s multiple comparison test. Differences among the CM differentiated monocytes or moMDSCs cocultured with CRC-PDTOs were performed using either a paired t-test or a Wilcoxon test. The statistical significance is depicted as follows: *p < 0.05, **p < 0.01, ***p < 0.001.

## Results

### Melanoma-derived PGE2 steers human monocytes towards an MDSC-like state

**Figure 1:**
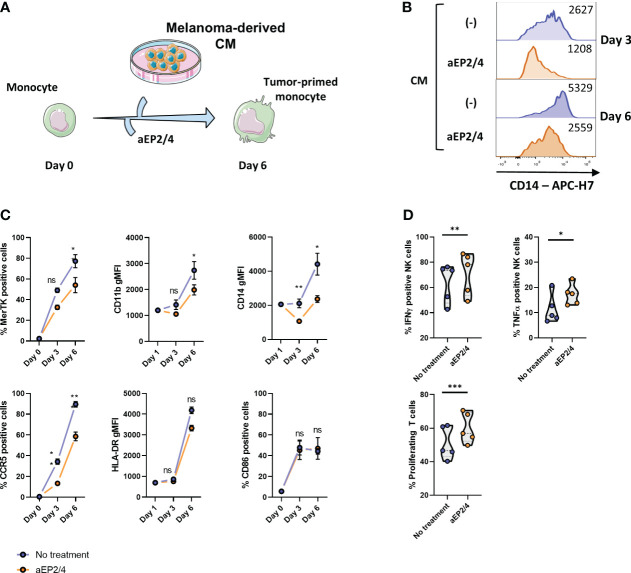
Tumor-derived PGE2 skews monocytes via EP2/EP4 receptors towards a suppressive state. **(A)** To examine the effect of PGE2-EP2/4 signaling during M-MDSC like cell development, monocytes were treated with aEP2/4 prior to exposure to melanoma cell line-derived CM. After day 3 and day 6 of treatment, the resulting cells were analyzed for the expression of MerTK, CD11b, CD14, CCR5, HLA-DR, and CD86. **(B)** Histograms showing the expression levels of CD14 of a representative donor with and without aEP2/4 treatment on day 3 and day 6. **(C)** Graphs show either the percentage of positive cells for MerTK, CCR5 and CD86, or the geometric mean fluorescence intensity (gMFI) of CD11b, CD14 and CCR5. Each dot represents the mean ± SD of monocytes from different donors (n≥3) combined from two independent experiments. **(D)** The functional relevance of targeting aEP2/4 was determined by coculturing the resulting M-MDSC-like cells with autologous T cells and NK cells. After three days, T cell proliferation was determined as loss of CFSE dye. For NK cell suppression, the intracellular levels of IFNγ and TNFα were determined. Violin plots show the mean, and each data point represents an individual monocyte donor (n=5) from a single independent experiment. P values were calculated with either a paired t-test or a Wilcoxon test (*p 0.05; **p 0.01; ***p 0.001). ns, non significant.

Monocytes exposed to tumor signals acquire a suppressive phenotype and function that recapitulate that of MDSCs (24, 25, 55). As model to determine the role of tumor-derived PGE2 signaling during monocyte development into MDSC-like cells, monocytes were cultured for 6 days with conditioned media (CM) derived from the melanoma cell line A375 (**Figure 1A**), detected to produce PGE2 (see **Supplementary Figure 1A**). To establish the effect of PGE2-EP2/4 signaling during MDSC-like differentiation, monocytes were incubated with specific antagonists for EP2 and EP4 (aEP2/4). Phenotype analysis after 3 and 6 days showed that EP2/4 blockade limited the expression of CD14, MerTK, CCR5, and CD11b (**Figures 1B, C**). No significant changes were detected for the markers HLA-DR and CD86. To assess the functional relevance of targeting PGE2-EP2/4 axis, monocytes cultured for 6 days in the presence of CM were harvested and cocultured with autologous NK and T cells. aEP2/4 attenuated the suppressive function of CM-educated monocytes as seen by the increased T cell proliferation and increased expression of the pro-inflammatory cytokines TNFa and IFNγ in NK cells (**Figure 1D**). Overall, these data illustrate the role PGE2-EP2/4 axis in the priming of a suppressive MDSC-like state of monocytes.

### PGE2-EP2/4 signaling mediates the occurrence of a suppressive moMDSC phenotype

**Figure 2:**
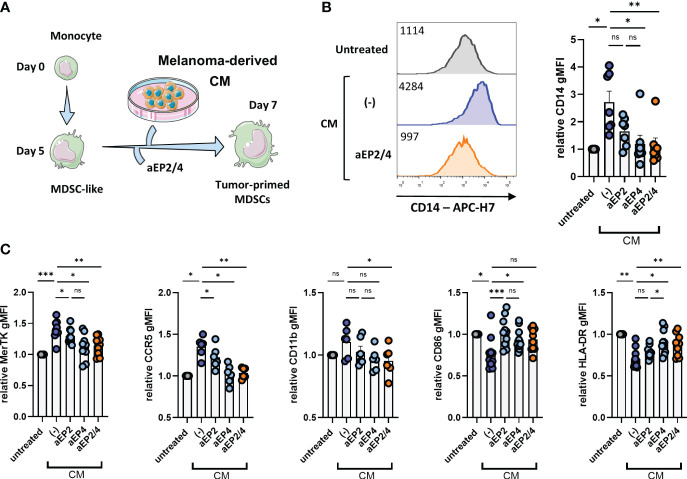
Tumor-derived PGE2 signaling mediates the occurrence of a suppressive moMDSC phenotype via EP2 and EP4. **(A)** *In vitro* generated moMDSCs were treated with aEP2, aEP4 or aEP2/4 prior to exposure to melanoma-derived CM. After 48 hours, moMDSCs were analyzed for the expression of CD14, MerTK, CCR5, CD11b, CD86, and HLA-DR by flow cytometry. **(B)** Representative histograms showing the expression levels of CD14 on untreated moMDSCs and moMDSCs treated with CM with or without aEP2/4 treatment. Graph represents the relative CD14 gMFI expression. Bar graph shows the mean ± SEM and each data point represents an individual M-MDSCs donor (n=7) tested in two independent experiments. **(C)** Bar graphs showing relative gMFI levels of MerTK, CCR5, CD11b, CD86, and HLA-DR. Relative gMFIs were determined by normalizing to gMFI levels of the untreated moMDSCs for every donor. Bar graphs show the mean ± SEM and each data point represents an individual moMDSCs donor (n>6) tested in two independent experiments. P values were calculated on raw gMFI values with either a Friedman test followed by a Dunn’s test or a one-way ANOVA with Tukey test for multiple comparison correction (*p 0.05; **p 0.01; ***p 0.001). ns, non significant.

PGE2 is well known as a modulator of the phenotype of myeloid cells, including different cell models of mouse and human MDSCs (23, 24, 53, 54). As a human M-MDSC model, we used moMDSCs, generated from monocytes by culturing them for 5 days in the presence of IL-6 and GM-CSF, previously reported to express typical MDSC markers such as CD11b or CD33 (21, 31). To distinguish the effects of tumor-derived PGE2 signaling trough the different PGE2 receptors, A375-derived CM was added to moMDSCs that were preincubated for 2 hours with aEP2, aEP4, or the combination designated as aEP2/4 (**Figure 2A**). After 48 hours, CM treatment led to the upregulation of CD14, MerTK, and CCR5, and the downregulation of HLA-DR and CD86 (**Figures 2B, C**). Although not significant, we noted a tendency towards the upregulation of CD11b. EP blockade demonstrated both EP2 and EP4 to contribute to the induction of this phenotype. Of note, aEP4 more robustly than aEP2 prevented the acquisition of this phenotype. Combined aEP2/4 blockade prevented the acquisition of the phenotype yielded by the tumor-derived CM on moMDSCs.

### PGE2-EP2/4 signaling induces the production of suppressive mediators by moMDSCs

**Figure 3:**
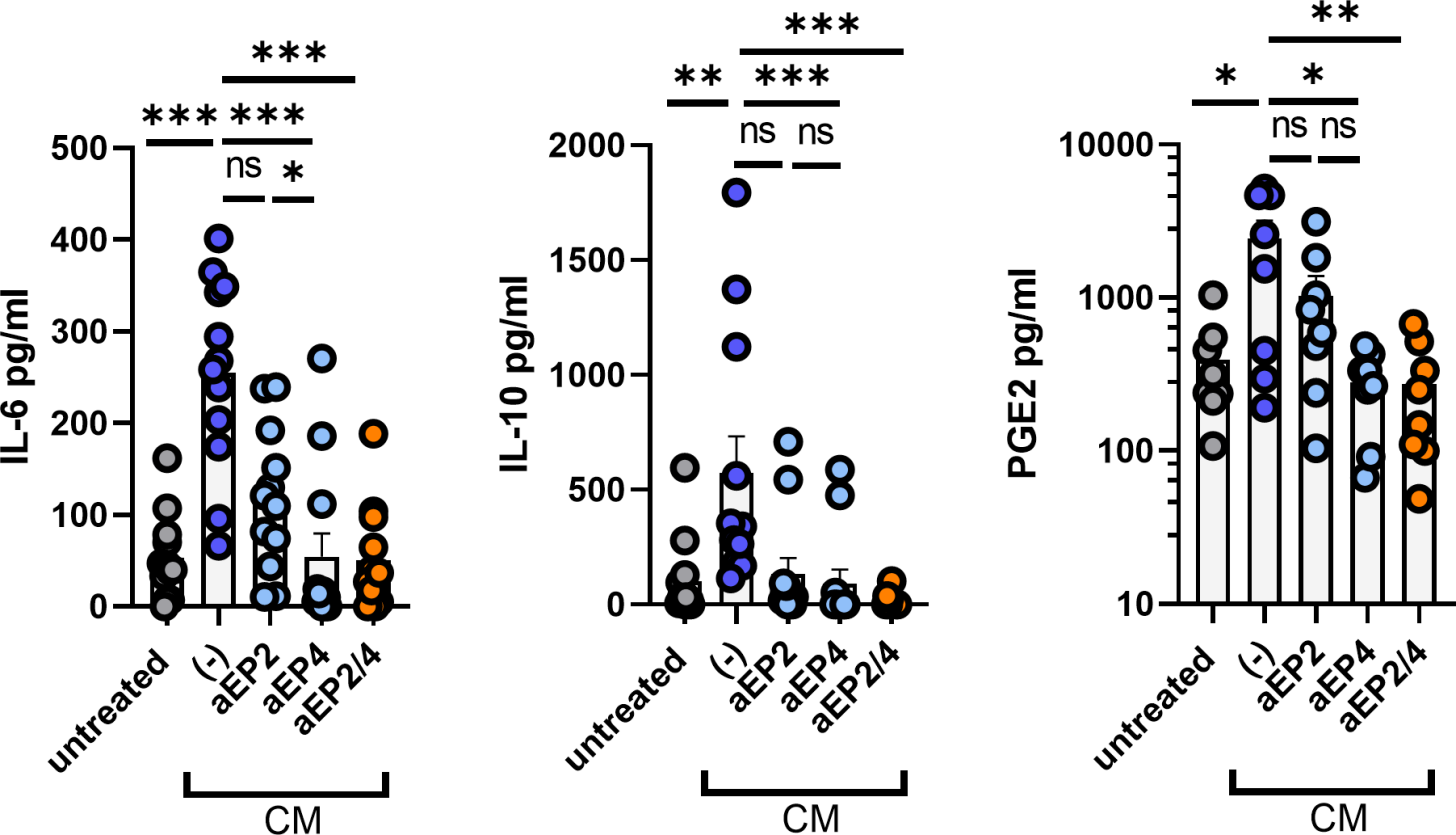
Tumor-derived PGE2 signaling induces the production of soluble factors on moMDSCs via EP2 and EP4. *In vitro* generated moMDSCs were treated with either aEP2, aEP4, or aEP2/4 prior to exposure to melanoma-derived CM. After 48 hours, moMDSCs were harvested, replated, and stimulated with 100 ng/ml of LPS. After overnight restimulation, moMDSCs supernatants were harvested and analyzed for the presence of the cytokines IL-6 and IL-10, and the lipid mediator PGE2 by ELISA. Data shows the pg/ml of each of these soluble factors. Bar graphs show the mean ± SEM and each data point represents an individual moMDSC donor (n>8) tested in three independent experiments. P values were calculated on raw concentration values with a Friedman test followed by a Dunn’s multiple comparison test (*p 0.05; **p 0.01; ***p 0.001). ns, non significant.

Suppressive M-MDSCs exploit a variety of mechanisms to establish a suppressive environment, such as the secretion of suppressive mediators (22). To determine the effect of PGE2-EP2/4 axis on the secretome of moMDSCs, we measured the levels of IL-6, IL-10, and PGE2. MoMDSCs exposed to CM displayed a robust upregulation of these three soluble mediators. aEP2 modestly inhibited the production of these mediators, whereas aEP4 significantly impaired their production (**Figure 3**). Similar to aEP4 treatment, the combined aEP2/4 inhibited the secretion of these three mediators, recapitulating the production levels of moMDSCs unexposed to CM. Overall, these results identify the PGE2-EP2/4 axis as a major modulator of IL-10, IL-6, and PGE2 production by moMDSCs, with EP4 more robustly than EP2 mediating this feature.

### PGE2-EP2/4 educated moMDSCs suppress autologous T cell responses

**Figure 4:**
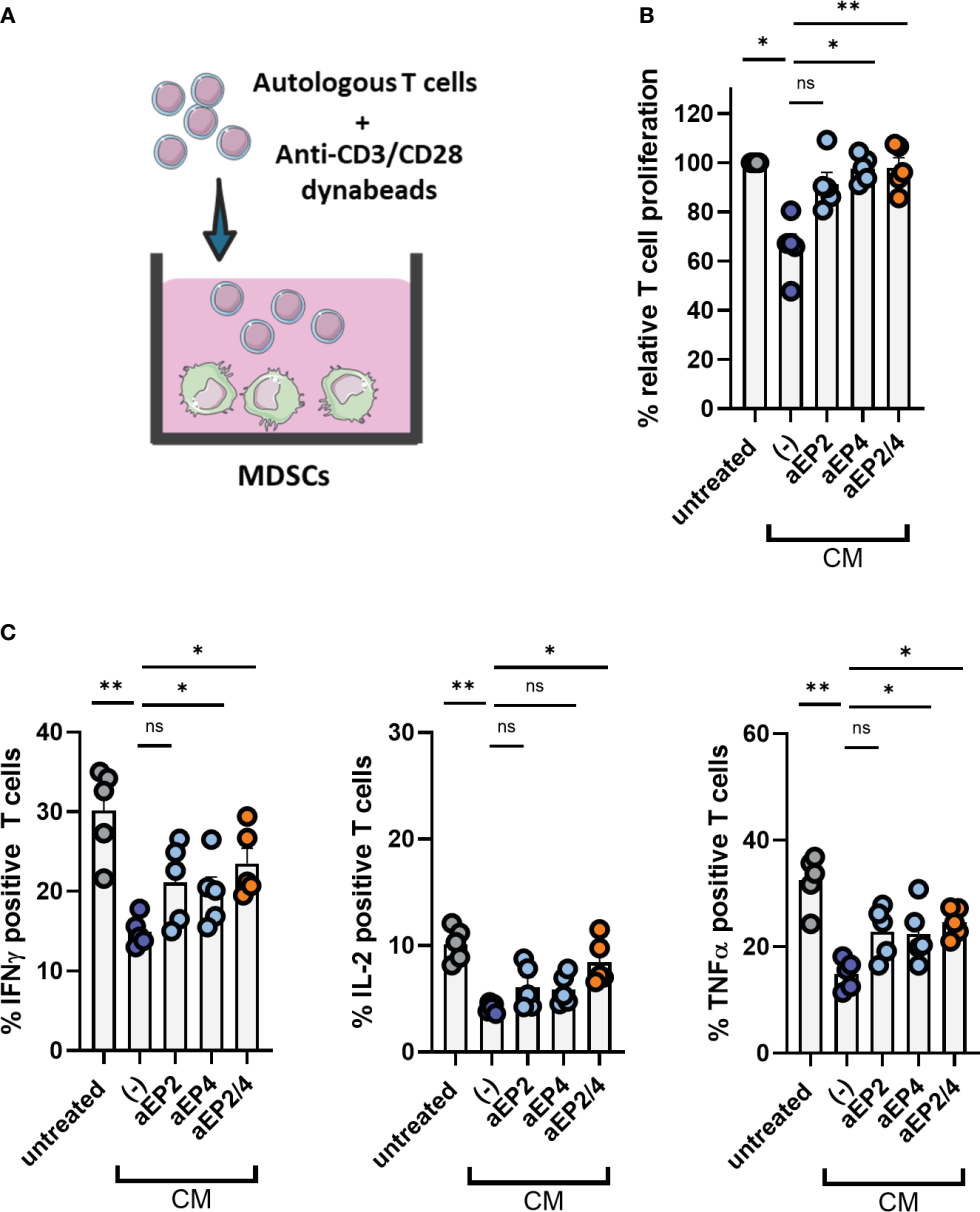
PGE2-educated moMDSCs suppress autologous T cell responses via EP2 and EP4. *In vitro* generated moMDSCs were treated with either aEP2, aEP4, or aEP2/4 prior to exposure to melanoma-derived CM. After 48 hours, moMDSCs were harvested and replated for posterior coculturing with T cells. **(A)** moMDSCs were cocultured with autologous T cells previously primed to proliferate using activating dynabeads (anti-CD3/CD28). **(B)** Bar graphs showing the relative T cell proliferation. Relative proliferation was determined by normalizing to the T cell proliferation given by untreated moMDSCs. Each data point represents an individual moMDSC donor (n=5) tested in two independent experiments. **(C)** Production of IFNγ, IL-2, and TNFα by T cells was determined by intracellular staining. Bar graphs show the percentage of T cells positive for the indicated cytokine. Each data point represents an individual moMDSC donor (n=5) from a single independent experiment. P values were calculated on raw percentage values with one-way ANOVA followed by a Dunnett’s comparison test (*p 0.05; **p 0.01; ***p 0.001). ns, non significant.

M-MDSCs are potent suppressors of T cell immune responses as they inhibit T cell proliferation and the production of pro-inflammatory cytokines such as IFNγ (20, 21, 24–26, 28). To assess the ability of moMDSCs to modulate T cell proliferation, moMDSCs were cocultured with autologous T cells labelled with CFSE dye for three days (**Figure 4A**). Additionally, moMDSCs were cocultured with T cells not labelled with CFSE for the intracellular detection of cytokines. Prior to coculture, T cells were primed to proliferate by using activating anti-CD3/CD28 dynabeads. Exposure to CM led to moMDSCs with a superior ability to suppress the proliferation of autologous T cells (**Figure 4B**). Cytokine analysis demonstrated that CM treated moMDSCs yielded T cells with a reduced production of IFNγ, TNFα, and IL-2, cytokines associated with active pro-inflammatory T cell responses (**Figure 4C**).

Given the strong phenotypical modulation of moMDSCs achieved by blocking EP2 and EP4, we further assessed whether this inhibition could recover T cell function upon moMDSC coculture. aEP2 tended to partly recover T cell proliferation, whereas aEP4 significantly restored T cell proliferation to the levels of moMDSCs unexposed to the CM. As expected, combined aEP2/4 restored T cell proliferation to a level similar to that induced by aEP4 (**Figure 4B**). Although not significantly, cytokine analysis showed both EP receptors similarly contribute to the downregulation of IFNγ, IL-2, and TNFα production by T cells after moMDSC coculture. The combined aEP2/4 further upregulated the expression of these cytokines compared to the individual blockade of EP2 and EP4 (**Figure 4C**). Further characterization of CD4 and CD8 T cells within the cocultures demonstrated these suppressive features to be similarly present across CD4 and CD8 T cells (see supplementary **Figures 3C, D**).

### PGE2-EP2/4 signaling potentiates the suppressive ability of moMDSCs over NK cells

**Figure 5:**
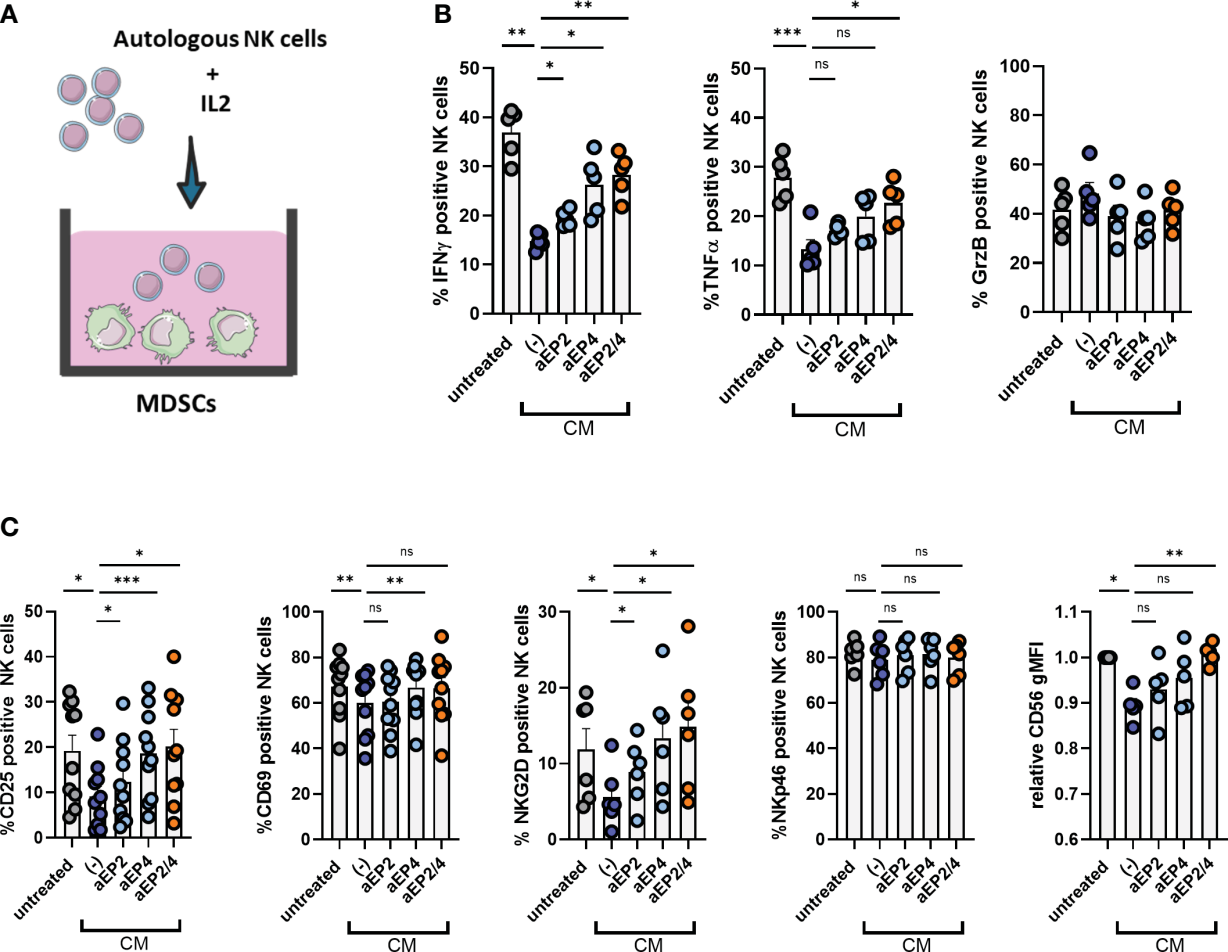
PGE2-EP2/4 signaling potentiates the NK cell-suppressive ability of moMDSCs. *In vitro* generated moMDSCs were treated with either aEP2, aEP4, or aEP2/4 prior to exposure to melanoma-derived CM. After 48 hours, moMDSCs were harvested and replated for coculturing with NK cells. **(A)** moMDSCs were cocultured with autologous NK cells preactivated with IL-2. **(B)** Production of IFNγ, TNFα, and GrzB by NK cells was determined by intracellular staining. Bar graphs are showing the percentage of positive NK cells for the indicated cytokine. Each data point represents and individual moMDSC donor (n=5) from a single independent experiment. **(C)** Bar graphs show the expression levels of different NK cell markers, either in percentage (NKG2D, NKp46, CD25, CD69) or relative gMFI (CD56). Relative CD56 gMFI expression was determined by normalizing to the expression levels of the untreated condition for every donor. Each data point represents an independent moMDSC donor (n>6) tested in two independent experiments. P values were calculated on the percentage or raw gMFI values with either a Friedman test followed by a Dunn’s test or a one-way ANOVA followed by a Dunnett’s comparison test (*p 0.05; **p 0.01; ***p 0.001). ns, non significant.

Tumor cell clearance requires active immune responses by cytotoxic lymphocytes, including NK cells. PGE2 signaling on M-MDSCs has demonstrated to mediate NK cell dysfunction in the tumor tissue, enabling tumor progression (28, 29). To investigate the relevance of M-MDSCs on NK cells, moMDSCs were cocultured with autologous NK cells pre-stimulated with IL-2 (**Figure 5A**). After three days, NK cells were characterized for the expression of different activation surface markers and the production of pro-inflammatory cytokines. CM treated moMDSCs suppressed the production of IFNγ and TNFα, whereas no changes were detected for granzyme B (**Figure 5B**). NK activation marker analysis showed CM-treated moMDSCs to suppress the expression of CD25, CD69, and NKG2D, whereas NKp46 levels remained unchanged (**Figure 5C**). Of note, a significant downregulation of the lineage marker CD56 on NK cells was observed. EP2 and EP4 modulation demonstrated aEP4 rather than aEP2 to primarily recover the expression of CD25, CD69, NKG2D, IFNγ, and TNFα. Combined aEP2/4 further recovered the expression of these markers compared to aEP4 only, resembling the NK suppression level induced by untreated moMDSCs. Overall, these results indicate the synergistic contribution of EP2 and EP4 on moMDSCs during NK cell suppression, with EP4 primarily mediating such feature.

### PGE2-EP2/4 signaling on moMDSCs steers moDC mediated T cell responses towards suppressive T cell populations

**Figure 6:**
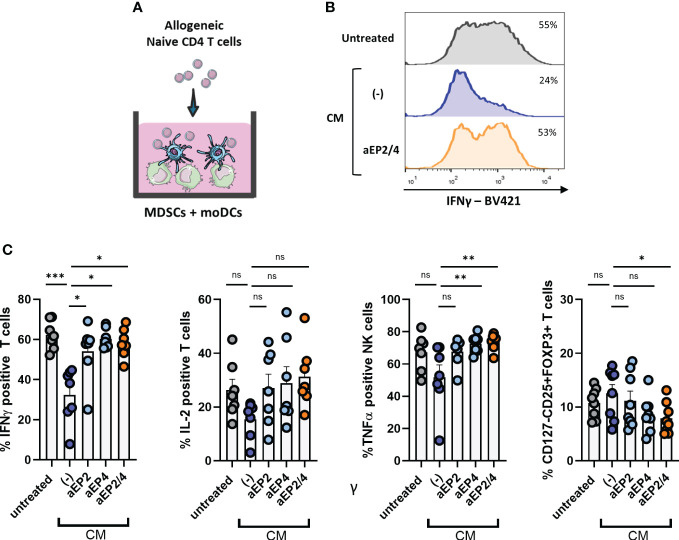
PGE2-EP2/4 signaling in moMDSCs steers moDC-mediated T cell responses towards the induction of suppressive T cell populations. **(A)** *In vitro* generated moMDSCs were treated with aEP2, aEP4, or aEP2/4 prior to exposure to melanoma-derived CM. After 48 hours, moMDSCs were harvested and replated for coculturing with moDCs and naïve CD4 T cells. To assess the impact of moMDSCs on the ability of moDCs to induce pro-inflammatory T cell responses, moMDSCs were cocultured with moDCs and allogeneic naïve CD4 T cells for 12 days. **(B)** Histograms show the intracellular IFNγ levels in T cells after coculture from a representative donor. **(C)** Percentage of T cells positive for intracellular IFNγ, IL-2, and TNFα expression. Bar graphs show the mean ± SEM and each data point represents an individual M-MDSCs donor (n=7) tested in two independent experiments. **(C)** T cells were analyzed for the expression of CD125, CD25, and FOXP3 to identify Tregs. Bar graphs show the percentage of CD127-CD25+FOXP3+ cells. Each data point represents expanded T cells from an individual moMDSC donor (n=8), derived from two independent experiments, and the bar graphs show the mean ± SEM. P values were calculated with either a Friedman test followed by a Dunn’s test or a one-way ANOVA with a Dunnett’s test for multiple comparison (*p 0.05; **p 0.01; ***p 0.001). ns, non significant.

DCs are key initiators of anti-tumor immunity via priming tumor antigen specific T cell responses (58). However, this anti-tumoricidal DC feature is often impaired in cancer patients due to the suppressive TME. Although this outcome likely arises from tumor-mediated DC dysfunction, we hypothesize that environmental M-MDSCs can further steer or compromise the development of pro-inflammatory T cell responses regardless of the activation status of DCs. To address this question, moMDSCs were cocultured with moDCs previously treated with poly I:C and allogeneic naïve CD4 T cells (**Figure 6A**). After coculture, the resulting T cell populations were analyzed for the expression of the pro-inflammatory cytokines IFNγ, TNFα, and IL-2 (**Figures 6B**, **6C**). Additionally, Treg occurrence was assessed by determining the expression of CD127, CD25, and FOXP3 (**Figure 6C**). T cell analysis showed CM-treated MDSCs in coculture with moDCs to robustly suppress IFNγ responses. Although not significant we observed a tendency towards the downregulation of TNFα and IL-2, and a modest increase in Tregs. EP2 and EP4 blockade demonstrated both receptors contribute to this phenotype, although we noted a higher EP4 contribution compared to that of EP2. Combined aEP2/4 on moMDSCs recovered IFNγ and TNFα production to the level of untreated moMDSCs. Additionally, aEP2/4 significantly diminished the enrichment of Tregs compared to CM treated moMDSCs. Collectively, these results show that PGE2-EP2/4 signaling in moMDSCs skew moDC-mediated T cell responses towards the expansion of T cells with a suppressive rather than a pro-inflammatory profile.

### EP2/4 blockade attenuates the suppressive phenotype of moMDSCs in 3D cocultures with colorectal cancer patient-derived tumor organoids

**Figure 7:**
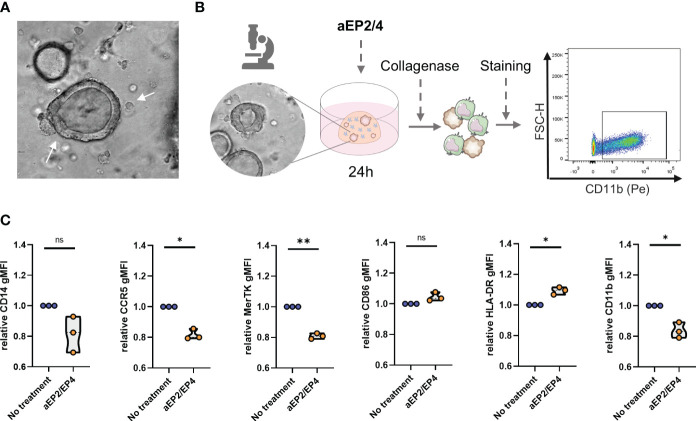
EP2/4 blockade attenuates the suppressive phenotype of moMDSCs in 3D cocultures with colorectal cancer patient derived tumor organoids (CRC-PDTO). **(A)** moMDSCs were cocultured with the CRC-PDTOs and treated with aEP2/4. **(B)** After 24 hours, the cocultures were dissociated and stained for flow cytometric analysis. moMDSCs identification in cocultures was assessed by gating on CD11b positive cells. **(C)** Bar graphs showing the relative gMFI expression levels of CD14, CCR5, MerTK, CD86, HLA-DR, and CD11b. Each data point represents an individual moMDSC donor (n=3) from a single independent experiment. P values were calculated with a paired t test (*p 0.05; **p 0.01; ***p 0.001).

To assess the impact of targeting EP2/4 signaling in a model that better recapitulates the TME, we performed a 3D coculture of moMDSCs with a CRC-PDTO derived from liver metastasis. MoMDSCs were co-cultured in a collagen matrix together with the CRC-PDTOs to allow PDTOs and moMDSCs interaction (**Figure 7A**). After 24 hours, cocultures were treated with aEP2/4. Next day, collagen cultures were dissociated, and the resulting cell suspension was stained and analyzed for flow cytometry. MoMDSCs were distinguished based on positivity for CD11b (**Figure 7B**). aEP2/4 treatment of the cocultured cells led to M-MDSCs with lower expression levels of CD11b, MerTK, and CCR5, and higher levels of CD86 (**Figure 7C**). Although not significant, we noted aEP2/4 tended to downregulate the expression of CD14. Overall, aEP2/4 can significantly attenuate the suppressive phenotype of moMDSCs cultured in a 3D TME model.

## Discussion

Tumor-derived PGE2 is well established as major factor mediating the development of MDSCs as well as boosting a pro-tumor phenotype and function (20, 24, 28, 34, 35). Yet, the phenotypical and functional implications of tumor-derived PGE2 signaling via either EP2 and EP4, or the relevance of targeting these receptors in human M-MDSCs exposed to tumor-derived PGE2 is poorly defined. In this study, we show that EP2 and EP4 contribute to the induction of a suppressive phenotype and function using an *in vitro* model for M-MDSCs. We show that the combined blockade of EP2 and EP4 attenuates a pro-tumor phenotype and function of moMDSCs exposed to tumor-derived PGE2 in 2D using melanoma CM and in a 3D coculture with CRC-PDTOs.

M-MDSCs in cancer patients are primarily derived from bone marrow precursors. However, cumulative evidence suggests that blood monocytes can also serve as precursors for M-MDSCs upon encountering tumor-derived signals (20, 24, 25, 33, 55). Exposure of freshly isolated monocytes to CM derived from the melanoma cell line A375 led to the acquisition of a pro-tumor state. In line with a prior study using a COX inhibitor (24), targeting EP2/4 signaling during monocyte differentiation limited the expression of suppressive markers, including CD14 or CD11b. We also noted an attenuation of the suppressive capacity of these cells, as seen by the increased T cell proliferation and superior expression of IFNγ and TNFα on NK cells. Noteworthy, CCR5 expression on MDSCs has been demonstrated to correlate with a superior suppressive capacity (59). In line with this publication, we noted that EP2/4 blockade attenuated the expression of CCR5 indeed correlating with a reduced suppressive capacity. Besides being indicative for the MDSC suppressive ability, CCR5 expression enables MDSC recruitment into tumor tissue. Knowing that PGE2 can be found at high levels in the blood of melanoma patients (data not shown) we hypothesize that circulating PGE2 might fine-tune blood monocytes to acquire a suppressive phenotype, mediate CCR5 expression, and facilitate their recruitment into the tumor tissue.

PGE2-EP2/4 signaling is involved not only in the differentiation of MDSCs, but also in the acquisition of a pro-tumoral suppressive phenotype (23, 24, 53, 54). In the here used human M-MDSC model, we observed that upon exposure to melanoma CM, moMDSCs further acquired a suppressive phenotype, characterized by the upregulation of CD14, CCR5, MerTK, and the downregulation of HLA-DR and CD86. Additionally, CM treatment boosted moMDSCs production of IL-6, IL-10, and PGE2, soluble mediators associated with tumor-promoting inflammation. EP2 and EP4 blockade demonstrated both receptors to contribute to the acquisition of this phenotype. However, we noted a superior EP4 contribution rather than that of EP2 for the acquisition of such features. These data are in line with previous mice studies where EP4 blockade prevented the downregulation of activating myeloid markers such as HLA-DR and CD86 on M-MDSCs (52, 53, 60), or hampered the expression of PGE2, IL-6, and IL-10 (53). Additionally, although there are limited studies conducted on human M-MDSCs, studies using moDCs have demonstrated that PGE2 via EP2 and EP4 boosts CD14, COX2, and IL-10 expression together with the downregulation of HLA-DR (20, 61), resembling the effect here reported for our *in vitro* M-MDSC model.

M-MDSCs display a variety of suppressive functional features, which hamper the induction of pro-inflammatory immune responses and skews the host immune system towards the acquisition of a tolerogenic and regulatory state, enabling tumor progression. These pleiotropic mechanisms of suppression are dependent on the abundance of different environmental tumor factors. In the here used M-MDSC model, we noted that providing melanoma CM to moMDSCs led to the suppression of autologous T cells. In line with prior studies (20, 21, 24, 25), coculture with autologous T cells demonstrated to limit T cell proliferation and to reduce the expression of pro-inflammatory cytokines such as IFNγ and TNFα. Additionally, we noted that CM-treated moMDSCs suppressed autologous NK cell responses. CM-treated moMDSCs led to the downregulation of NK cell activation markers including NKG2D, CD25, CD69 and the expression of the cytokines IFNγ and TNFα after co-culture with M-MDSCs. Addition of M-MDSC to moDCs and naïve CD4 T cell cocultures yielded T cells with reduced production of IFNγ and TNFα. Targeting PGE2-EP2/4 axis on moMDSCs successfully recovered the moDC-induced T cell and NK cell responses. In these cultures, a modest reduction of the Treg was observed upon EP2/4 blockade, aligning with a previous mice study (53). PGE2 involvement in the suppression of T and NK cell responses has been previously reported for mice MDSCs and human moDCs, where PGE2 primed MDSC-like features (20, 28, 50, 53, 61). Although we noted that T- and NK cell responses were reversed either partly (when individually targeting EP2 and EP4) or completely (when simultaneously targeting EP2 and EP4), we noted a different EP implication, with EP4 rather than EP2 primarily mediating these suppressive features of moMDSCs.

The predominant involvement of EP4 for the prevention of PGE2-mediated M-MDSC suppression of T cells and NK cells has already been demonstrated for mice MDSCs (28, 52, 54). Although EP4 targeting in mice displays a therapeutic benefit in different tumor models, few studies have explored the potential benefit of targeting EP2, EP4, or both. For example, two studies in mice demonstrated EP2 next to EP4 targeting to impair the development of MDSCs and limit tumor growth (49, 50). A recent publication exploring a dual EP2/4 antagonist has demonstrated to outperform the individual targeting of EP4 or EP2 for immunomodulation, hence preventing tumor growth (62, 63). The limited available studies with human cells use monocytes and moDCs demonstrate a similar contribution of EP2 and EP4 during the acquisition of MDSC-like features (20, 28, 53). Noteworthy, a recent study demonstrated that EP2 is expressed on blood MDSCs from colorectal cancer patients (50), underscoring the potential functional implication of EP2. We thus hypothesize that human MDSCs rely on both EP2 and EP4 signaling for acquiring a fully suppressive state.

To further evaluate the relevance of targeting EP2/4 signaling in a tumor model that better recapitulates the TME, we cocultured moMDSCs with CRC-PDTO. Notably, the combined application of EP2/4 antagonists within the coculture attenuated the development of a suppressive phenotype, as seen by the reduced CD11b, MerTK or CCR5 expression. Based on our observations and current literature, we propose that both EP2 and EP4 fosters human M-MDSC transition towards a suppressive pro-tumor state able to dampen T cell and NK cell responses, arguing for the combined usage of EP2 and EP4 antagonists to prevent a tumor-derived PGE2 imprinted phenotype of M-MDSCs. Although we show here the key role of EP2 and EP4 in *in vitro* generated human M-MDSCs, future research is required to validate the EP2/4 relevance on other blood MDSCs subsets. In different types of tumors, PMN-MDSCs, instead of the here investigated M-MDSCs model, have been reported to play a major role in promoting a suppressive TME. Unlike M-MDSCs, there are currently no cell models to study human blood PMN-MDSCs. Altogether, further exploration of PGE2-EP2/4 axis on blood MDSC subsets could serve to further establish the potential of targeting EP2/4 for therapeutic intervention in cancer patients.

Prior studies have demonstrated that PGE2-EP2/4 targeting can modulate immune cell function in different tumor models and enhance anti-tumor immunity (62–64). This illustrates the potential of targeting EP2/4 for the treatment of cancer. In fact, there are several ongoing phase I clinical trials for the treatment of cancer, either targeting EP4 or the combined EP2/4 (NCT04344795, NCT03155061) (65, 66). Using an *in vitro* cell model for M-MDSCs, our results further support the evidence that targeting EP2 and EP4 possess a therapeutic value for the immune modulation of the host immune system, alleviate tumor-derived suppression and thus, promote the development of anti-tumor immunity.

## Data availability statement

The raw data supporting the conclusions of this article will be made available by the authors, without undue reservation.

## Ethics statement

The studies involving humans were approved by Institutional Review Board of the Radboudumc (2005-03-TOR-TIL). The studies were conducted in accordance with the local legislation and institutional requirements. The participants provided their written informed consent to participate in this study.

## Author contributions

JC-E: Conceptualization, Investigation, Writing – original draft. BS: Investigation, Writing – review & editing. AG-P: Investigation, Writing – review & editing. AC: Supervision, Writing – review & editing. IJMdV: Supervision, Writing – review & editing. GF-G: Supervision, Writing – review & editing.

## References

[1] Mantovani A Allavena P Sica A Balkwill F. Cancer-related inflammation. Nature. (2008) 454(7203):436–44. doi: 10.1038/nature07205 18650914

[2] Gajewski TF Schreiber H Fu YX. Innate and adaptive immune cells in the tumor microenvironment. Nat Immunol (2013) 14(10):1014–22. doi: 10.1038/ni.2703 PMC4118725 24048123

[3] Hanahan D Weinberg RA. Hallmarks of cancer: the next generation. Cell. (2011) 144(5):646–74. doi: 10.1016/j.cell.2011.02.013 21376230

[4] Palucka AK Coussens LM. The basis of oncoimmunology. Cell. (2016) 164(6):1233–47. doi: 10.1016/j.cell.2016.01.049 PMC4788788 26967289

[5] Van Wigcheren GF De Haas N Mulder TA Horrevorts SK Bloemendal M Hins-Debree S. Cisplatin inhibits frequency and suppressive activity of monocytic myeloid-derived suppressor cells in cancer patients. Oncoimmunology. (2021) 10(1). doi: 10.1080/2162402X.2021.1935557 PMC8237969 34239773

[6] Diaz-Montero CM Salem ML Nishimura MI Garrett-Mayer E Cole DJ Montero AJ. Increased circulating myeloid-derived suppressor cells correlate with clinical cancer stage, metastatic tumor burden, and doxorubicin–cyclophosphamide chemotherapy. Cancer Immunology Immunother (2009) 58(1):49–59. doi: 10.1007/s00262-008-0523-4 PMC3401888 18446337

[7] Gordon IO Freedman RS. Defective antitumor function of monocyte-derived macrophages from epithelial ovarian cancer patients. Clin Cancer Res (2006) 12(5):1515–24. doi: 10.1158/1078-0432.CCR-05-2254 16533776

[8] Morse MA Hall JR Plate JM. Countering tumor-induced immunosuppression during immunotherapy for pancreatic cancer. Expert Opin Biol Ther (2009) 9(3):331–9. doi: 10.1517/14712590802715756 19216622

[9] Hoechst B Ormandy LA Ballmaier M Lehner F Krüger C Manns MP. A new population of myeloid-derived suppressor cells in hepatocellular carcinoma patients induces CD4+CD25+Foxp3+ T cells. Gastroenterology. (2008) 135(1):234–43. doi: 10.1053/j.gastro.2008.03.020 18485901

[10] Hoechst B Voigtlaender T Ormandy L Gamrekelashvili J Zhao F Wedemeyer H. Myeloid derived suppressor cells inhibit natural killer cells in patients with hepatocellular carcinoma via the NKp30 receptor. Hepatology. (2009) 50(3):799–807. doi: 10.1002/hep.23054 19551844 PMC6357774

[11] Filipazzi P Valenti R Huber V Pilla L Canese P Iero M. Identification of a new subset of myeloid suppressor cells in peripheral blood of melanoma patients with modulation by a granulocyte-macrophage colony-stimulation factor–based antitumor vaccine. J Clin Oncol (2007) 25(18):2546–53. doi: 10.1200/JCO.2006.08.5829 17577033

[12] Mandruzzato S Solito S Falisi E Francescato S Chiarion-Sileni V Mocellin S. IL4Rα+ Myeloid-derived suppressor cell expansion in cancer patients. J Immunol (2009) 182(10):6562–8. doi: 10.4049/jimmunol.0803831 19414811

[13] Cui TX Kryczek I Zhao L Zhao E Kuick R Roh MH. Myeloid-derived suppressor cells enhance stemness of cancer cells by inducing microRNA101 and suppressing the corepressor ctBP2. Immunity. (2013) 39(3):611–21. doi: 10.1016/j.immuni.2013.08.025 PMC3831370 24012420

[14] Bronte V Brandau S Chen SH Colombo MP Frey AB Greten TF. Recommendations for myeloid-derived suppressor cell nomenclature and characterization standards. Nat Commun (2016) 7(1):12150. doi: 10.1038/ncomms12150 27381735 PMC4935811

[15] Mandruzzato S Brandau S Britten CM Bronte V Damuzzo V Gouttefangeas C. Toward harmonized phenotyping of human myeloid-derived suppressor cells by flow cytometry: results from an interim study. Cancer Immunol Immunother. (2016) 65(2):161–9. doi: 10.1007/s00262-015-1782-5 PMC4726716 26728481

[16] Ochoa AC Zea AH Hernandez C Rodriguez PC. Arginase, prostaglandins, and myeloid-derived suppressor cells in renal cell carcinoma. Clin Cancer Res (2007) 13(2):721s–6s. doi: 10.1158/1078-0432.CCR-06-2197 17255300

[17] Mellor AL Munn DH. Ido expression by dendritic cells: tolerance and tryptophan catabolism. Nat Rev Immunol (2004) 4(10):762–74. doi: 10.1038/nri1457 15459668

[18] Bronte V Zanovello P. Regulation of immune responses by L-arginine metabolism. Nat Rev Immunol (2005) 5(8):641–54. doi: 10.1038/nri1668 16056256

[19] Sinha P Clements VK Bunt SK Albelda SM Ostrand-Rosenberg S. Cross-talk between myeloid-derived suppressor cells and macrophages subverts tumor immunity toward a type 2 response. J Immunol (2007) 179(2):977–83. doi: 10.4049/jimmunol.179.2.977 17617589

[20] Obermajer N Muthuswamy R Lesnock J Edwards RP Kalinski P. Positive feedback between PGE2 and COX2 redirects the differentiation of human dendritic cells toward stable myeloid-derived suppressor cells. Blood. (2011) 118(20):5498–505. doi: 10.1182/blood-2011-07-365825 PMC3217352 21972293

[21] van Wigcheren GF Cuenca-Escalona J Stelloo S Brake J Peeters E Horrevorts Sophie K. Myeloid-derived suppressor cells and tolerogenic dendritic cells are distinctively induced by PI3K and Wnt signaling pathways. J Biol Chem (2023) 299:105276. doi: 10.1016/j.jbc.2023.105276 37739035 PMC10628850

[22] Ostrand-Rosenberg S. Myeloid-derived suppressor cells: more mechanisms for inhibiting antitumor immunity. Cancer Immunology Immunother (2010) 59(10):1593–600. doi: 10.1007/s00262-010-0855-8 PMC3706261 20414655

[23] Poschke I Mougiakakos D Hansson J Masucci GV Kiessling R. Immature immunosuppressive CD14+HLA-DR–/low cells in melanoma patients are stat3hi and overexpress CD80, CD83, and DC-sign. Cancer Res (2010) 70(11):4335–45. doi: 10.1158/0008-5472.CAN-09-3767 20484028

[24] Mao Y Poschke I Wennerberg E Pico de Coaña Y Egyhazi Brage S Schultz I. Melanoma-educated CD14+ Cells acquire a myeloid-derived suppressor cell phenotype through COX-2–dependent mechanisms. Cancer Res (2013) 73(13):3877–87. doi: 10.1158/0008-5472.CAN-12-4115 23633486

[25] Lechner MG Megiel C Russell SM Bingham B Arger N Woo T. Functional characterization of human Cd33+ And Cd11b+ myeloid-derived suppressor cell subsets induced from peripheral blood mononuclear cells co-cultured with a diverse set of human tumor cell lines. J Transl Med (2011) 9(1):90. doi: 10.1186/1479-5876-9-90 21658270 PMC3128058

[26] Kusmartsev S Nagaraj S Gabrilovich DI. Tumor-associated CD8+ T cell tolerance induced by bone marrow-derived immature myeloid cells. J Immunol (2005) 175(7):4583–92. doi: 10.4049/jimmunol.175.7.4583 PMC1350970 16177103

[27] Bronte V Serafini P Apolloni E Zanovello P. Tumor-induced immune dysfunctions caused by myeloid suppressor cells. J Immunother (2001) 24(6):431–46. doi: 10.1097/00002371-200111000-00001 11759067

[28] Mao Y Sarhan D Steven A Seliger B Kiessling R Lundqvist A. Inhibition of tumor-derived prostaglandin-E2 blocks the induction of myeloid-derived suppressor cells and recovers natural killer cell activity. Clin Cancer Res (2014) 20(15):4096–106. doi: 10.1158/1078-0432.CCR-14-0635 24907113

[29] Li H Han Y Guo Q Zhang M Cao X. Cancer-expanded myeloid-derived suppressor cells induce anergy of NK cells through membrane-bound TGF-β1. J Immunol (2009) 182(1):240–9. doi: 10.4049/jimmunol.182.1.240 19109155

[30] Serafini P Mgebroff S Noonan K Borrello I. Myeloid-derived suppressor cells promote cross-tolerance in B-cell lymphoma by expanding regulatory T cells. Cancer Res (2008) 68(13):5439–49. doi: 10.1158/0008-5472.CAN-07-6621 PMC2887390 18593947

[31] Tomić S Joksimović B Bekić M Vasiljević M Milanović M Čolić M. Prostaglanin-E2 potentiates the suppressive functions of human mononuclear myeloid-derived suppressor cells and increases their capacity to expand IL-10-producing regulatory T cell subsets. Front Immunol (2019) 10. doi: 10.3389/fimmu.2019.00475 PMC6431635 30936876

[32] Lechner MG Liebertz DJ Epstein AL. Characterization of cytokine-induced myeloid-derived suppressor cells from normal human peripheral blood mononuclear cells. J Immunol (2010) 185(4):2273–84. doi: 10.4049/jimmunol.1000901 PMC2923483 20644162

[33] Casacuberta-Serra S Parés M Golbano A Coves E Espejo C Barquinero J. Myeloid-derived suppressor cells can be efficiently generated from human hematopoietic progenitors and peripheral blood monocytes. Immunol Cell Biol (2017) 95(6):538–48. doi: 10.1038/icb.2017.4 28108746

[34] Veltman JD Lambers ME van Nimwegen M Hendriks RW Hoogsteden HC Aerts JG. COX-2 inhibition improves immunotherapy and is associated with decreased numbers of myeloid-derived suppressor cells in mesothelioma. Celecoxib influences MDSC Funct BMC Cancer. (2010) 10(1):464. doi: 10.1186/1471-2407-10-464 PMC2939552 20804550

[35] Fujita M Kohanbash G Fellows-Mayle W Hamilton RL Komohara Y Decker SA. COX-2 blockade suppresses gliomagenesis by inhibiting myeloid-derived suppressor cells. Cancer Res (2011) 71(7):2664–74. doi: 10.1158/0008-5472.CAN-10-3055 PMC3075086 21324923

[36] Simmons DL Botting RM Hla T. Cyclooxygenase isozymes: the biology of prostaglandin synthesis and inhibition. Pharmacol Rev (2004) 56(3):387–437. doi: 10.1124/pr.56.3.3 15317910

[37] Ricciotti E FitzGerald GA. Prostaglandins and inflammation. Arterioscler Thromb Vasc Biol (2011) 31(5):986–1000. doi: 10.1161/ATVBAHA.110.207449 21508345 PMC3081099

[38] Kalinski P. Regulation of immune responses by prostaglandin E2. J Immunol (2012) 188(1):21–8. doi: 10.4049/jimmunol.1101029 PMC3249979 22187483

[39] Mizuno R Kawada K Sakai Y. Prostaglandin E2/EP signaling in the tumor microenvironment of colorectal cancer. Int J Mol Sci (2019) 20(24):6254. doi: 10.3390/ijms20246254 31835815 PMC6940958

[40] Ma S Guo C Sun C Han T Zhang H Qu G. Aspirin use and risk of breast cancer: A meta-analysis of observational studies from 1989 to 2019. Clin Breast Cancer. (2021) 21(6):552–65. doi: 10.1016/j.clbc.2021.02.005 33741292

[41] Sivak-Sears NR Schwartzbaum JA Miike R Moghadassi M Wrensch M. Case-control study of use of nonsteroidal antiinflammatory drugs and glioblastoma multiforme. Am J Epidemiol. (2004) 159(12):1131–9. doi: 10.1093/aje/kwh153 15191930

[42] Clouser MC Roe DJ Foote JA Harris RB. Effect of non-steroidal anti-inflammatory drugs on non-melanoma skin cancer incidence in the SKICAP-AK trial. Pharmacoepidemiol Drug Saf. (2009) 18(4):276–83. doi: 10.1002/pds.1718 19226541

[43] Baandrup L Faber MT Christensen J Jensen A Andersen KK Friis S. Nonsteroidal anti-inflammatory drugs and risk of ovarian cancer: systematic review and meta-analysis of observational studies. Acta Obstet Gynecol Scand (2013) 92(3):245–55. doi: 10.1111/aogs.12069 23240575

[44] Solomon SD McMurray JJV Pfeffer MA Wittes J Fowler R Finn P. Cardiovascular risk associated with celecoxib in a clinical trial for colorectal adenoma prevention. New Engl J Med (2005) 352(11):1071–80. doi: 10.1056/NEJMoa050405 15713944

[45] Bertagnolli MM Eagle CJ Zauber AG Redston M Breazna A Kim K. Five-year efficacy and safety analysis of the adenoma prevention with celecoxib trial. Cancer Prev Res (2009) 2(4):310–21. doi: 10.1158/1940-6207.CAPR-08-0206 PMC2976587 19336730

[46] Kawahara K Hohjoh H Inazumi T Tsuchiya S Sugimoto Y. Prostaglandin E2-induced inflammation: Relevance of prostaglandin E receptors. Biochim Biophys Acta (BBA) - Mol Cell Biol Lipids. (2015) 1851(4):414–21. doi: 10.1016/j.bbalip.2014.07.008 25038274

[47] Sugimoto Y Narumiya S. Prostaglandin E receptors. J Biol Chem (2007) 282(16):11613–7. doi: 10.1074/jbc.R600038200 17329241

[48] Harizi H Grosset C Gualde N. Prostaglandin E2 modulates dendritic cell function via EP2 and EP4 receptor subtypes. J Leukoc Biol (2003) 73(6):756–63. doi: 10.1189/jlb.1002483 12773508

[49] Sinha P Clements VK Fulton AM Ostrand-Rosenberg S. Prostaglandin E2 promotes tumor progression by inducing myeloid-derived suppressor cells. Cancer Res (2007) 67(9):4507–13. doi: 10.1158/0008-5472.CAN-06-4174 17483367

[50] Porta C Consonni FM Morlacchi S Sangaletti S Bleve A Totaro MG. Tumor-derived prostaglandin E2 promotes p50 NF-κB-dependent differentiation of monocytic MDSCs. Cancer Res (2020) 80(13):2874–88. doi: 10.1158/0008-5472.CAN-19-2843 32265223

[51] van Geffen C Deißler A Beer-Hammer S Nürnberg B Handgretinger R Renz H. Myeloid-derived suppressor cells dampen airway inflammation through prostaglandin E2 receptor 4. Front Immunol (2021) 12. doi: 10.3389/fimmu.2021.695933 PMC8311661 34322123

[52] Wang Y Cui L Georgiev P Singh L Zheng Y Yu Y. Combination of EP _4_ antagonist MF-766 and anti-PD-1 promotes anti-tumor efficacy by modulating both lymphocytes and myeloid cells. Oncoimmunology. (2021) 10(1). doi: 10.1080/2162402X.2021.1896643 PMC7993229 33796403

[53] Albu DI Wang Z Huang KC Wu J Twine N Leacu S. EP4 Antagonism by E7046 diminishes Myeloid immunosuppression and synergizes with Treg-reducing IL-2-Diphtheria toxin fusion protein in restoring anti-tumor immunity. Oncoimmunology. (2017) 6(8):e1338239. doi: 10.1080/2162402X.2017.1338239 28920002 PMC5593700

[54] Xu Y Zhao W Xu J Li J Hong Z Yin Z. Activated hepatic stellate cells promote liver cancer by induction of myeloid-derived suppressor cells through cyclooxygenase-2. Oncotarget. (2016) 7(8):8866–78. doi: 10.18632/oncotarget.6839 PMC4891010 26758420

[55] Okada SL Simmons RM Franke-Welch S Nguyen TH Korman AJ Dillon SR. Conditioned media from the renal cell carcinoma cell line 786.O drives human blood monocytes to a monocytic myeloid-derived suppressor cell phenotype. Cell Immunol (2018) 323:49–58. doi: 10.1016/j.cellimm.2017.10.014 29103587

[56] Iyer KK Poel D. High-dose short-term osimertinib treatment is effective in patient-derived metastatic colorectal cancer organoids. (2023), 1–25. doi: 10.21203/rs.3.rs-2867114/v1 PMC11523998 39516561

[57] Subtil B Iyer KK Poel D Gorris MAJ Cuenca J Cambi A. Dendritic cell phenotype and function in a 3D co-culture model of patient-derived metastatic colorectal cancer organoids. Front Immunol (2023) 14:1–13. doi: 10.3389/fimmu.2023.1105244 PMC9905679 36761758

[58] Kvedaraite E Ginhoux F. Human dendritic cells in cancer. Sci Immunol (2022) 7(70). doi: 10.1126/sciimmunol.abm9409 35363544

[59] Blattner C Fleming V Weber R Himmelhan B Altevogt P Gebhardt C. CCR5+ Myeloid-derived suppressor cells are enriched and activated in melanoma lesions. Cancer Res (2018) 78(1):157–67. doi: 10.1158/0008-5472.CAN-17-0348 29089297

[60] Karavitis J Hix LM Shi YH Schultz RF Khazaie K Zhang M. Regulation of COX2 expression in mouse mammary tumor cells controls bone metastasis and PGE2-induction of regulatory T cell migration. PloS One (2012) 7(9):e46342. doi: 10.1371/journal.pone.0046342 23029485 PMC3460819

[61] Rodríguez-Ubreva J Català-Moll F Obermajer N Álvarez-Errico D Ramirez RN Company C. Prostaglandin E2 leads to the acquisition of DNMT3A-dependent tolerogenic functions in human myeloid-derived suppressor cells. Cell Rep (2017) 21(1):154–67. doi: 10.1016/j.celrep.2017.09.018 28978469

[62] Francica BJ Holtz A Lopez J Freund D Chen A Wang D. Dual blockade of EP2 and EP4 signaling is required for optimal immune activation and antitumor activity against prostaglandin-expressing tumors. Cancer Res Commun (2023) 3(8):1486–500. doi: 10.1158/2767-9764.CRC-23-0249 PMC10408683 37559947

[63] Thumkeo D Punyawatthananukool S Prasongtanakij S Matsuura R Arima K Nie H. PGE2-EP2/EP4 signaling elicits immunosuppression by driving the mregDC-Treg axis in inflammatory tumor microenvironment. Cell Rep (2022) 39(10):110914. doi: 10.1016/j.celrep.2022.110914 35675777

[64] Bayerl F Meiser P Donakonda S Hirschberger A Lacher SB Pedde AM. Tumor-derived prostaglandin E2 programs cDC1 dysfunction to impair intratumoral orchestration of anti-cancer T cell responses. Immunity. (2023) 56(6):1341–1358.e11. doi: 10.1016/j.immuni.2023.05.011 37315536

[65] Davar D Powderly JD Ulahannan SV Johnson ML Sharma M Krauss JC. A phase 1 study of TPST-1495 as a single agent and in combination with pembrolizumab in subjects with solid tumors. J Clin Oncol (2022) 40(16_suppl):TPS2696–TPS2696. doi: 10.1200/JCO.2022.40.16_suppl.TPS2696

[66] Hong DS Parikh A Shapiro GI Varga A Naing A Meric-Bernstam F. First-in-human phase I study of immunomodulatory E7046, an antagonist of PGE _2_ -receptor E-type 4 (EP4), in patients with advanced cancers. J Immunother Cancer. (2020) 8(1):e000222. doi: 10.1136/jitc-2019-000222 32554609 PMC7304851

