# Supplementary material for: EP2 and EP4 blockade prevents tumor-induced suppressive features in human monocytic myeloid-derived suppressor cells
Source: Front Immunol. 2024 Jan 26;15:1355769. doi: 10.3389/fimmu.2024.1355769 (PMC10853404; doi:10.3389/fimmu.2024.1355769)
Supplement: Supplementary file 1 [file DataSheet_1.pdf]

Supplementary Material

Supplementary Figures

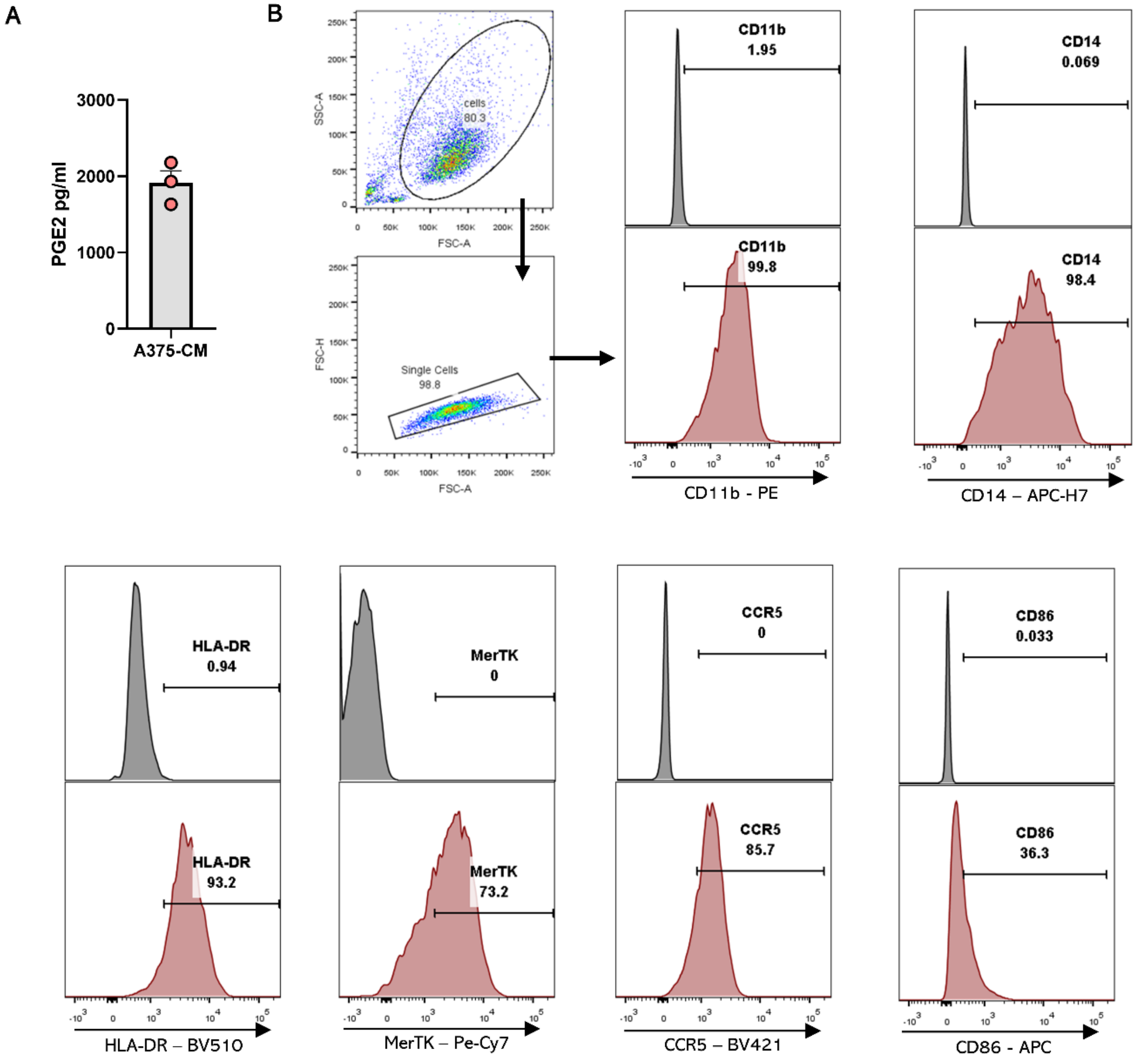

Supplementary Figure 1. PGE2 content in the melanoma A375 cell line CM and gating strategy to assess the phenotype of monocytes cocultured with melanoma derived CM. (A) PGE2 levels detected by ELISA in the A375 derived CM. (B) Gating strategy of CM educated monocytes by an initial gate based on the physical parameters FCS-A and SSC-A, followed by a second gate on single cells established using FCS-A and FCS-H. Expression of the indicated myeloid markers was determined by comparing stained monocytes cocultured with CM (depicted as a red histogram) with unstained cells for the indicated fluorochrome (depicted as the grey histogram).

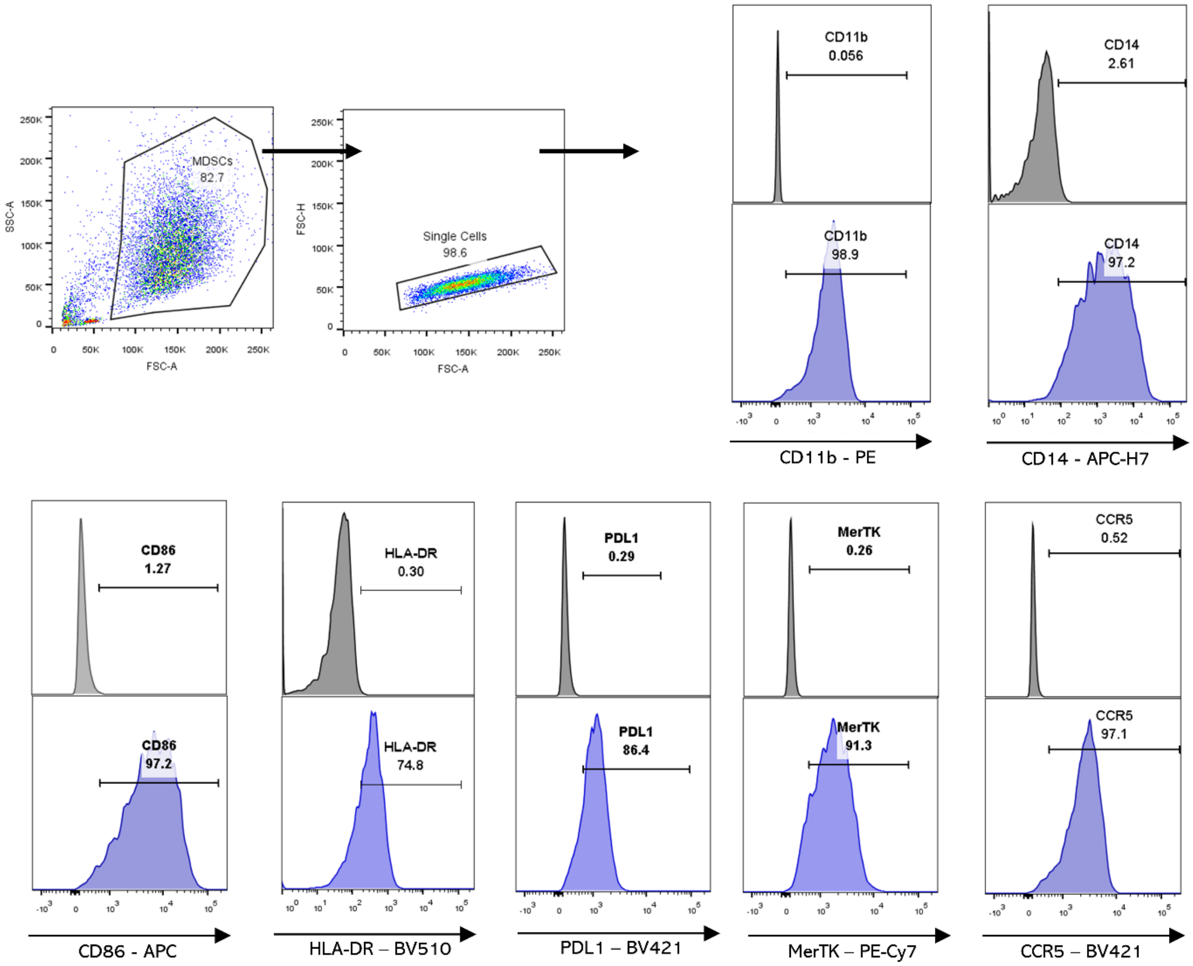

Supplementary Figure 2. Gating strategy to assess the phenotype of moMDSCs. An initial gate is set based on the physical parameters FCS-A and SSC-A, followed by a second gate on single cells using FCS-A and FCS-H. Expression of the indicated myeloid markers was determined by comparing stained MDSCs (depicted as a blue histogram) with unstained cells for the indicated fluorochrome (depicted as the grey histogram).

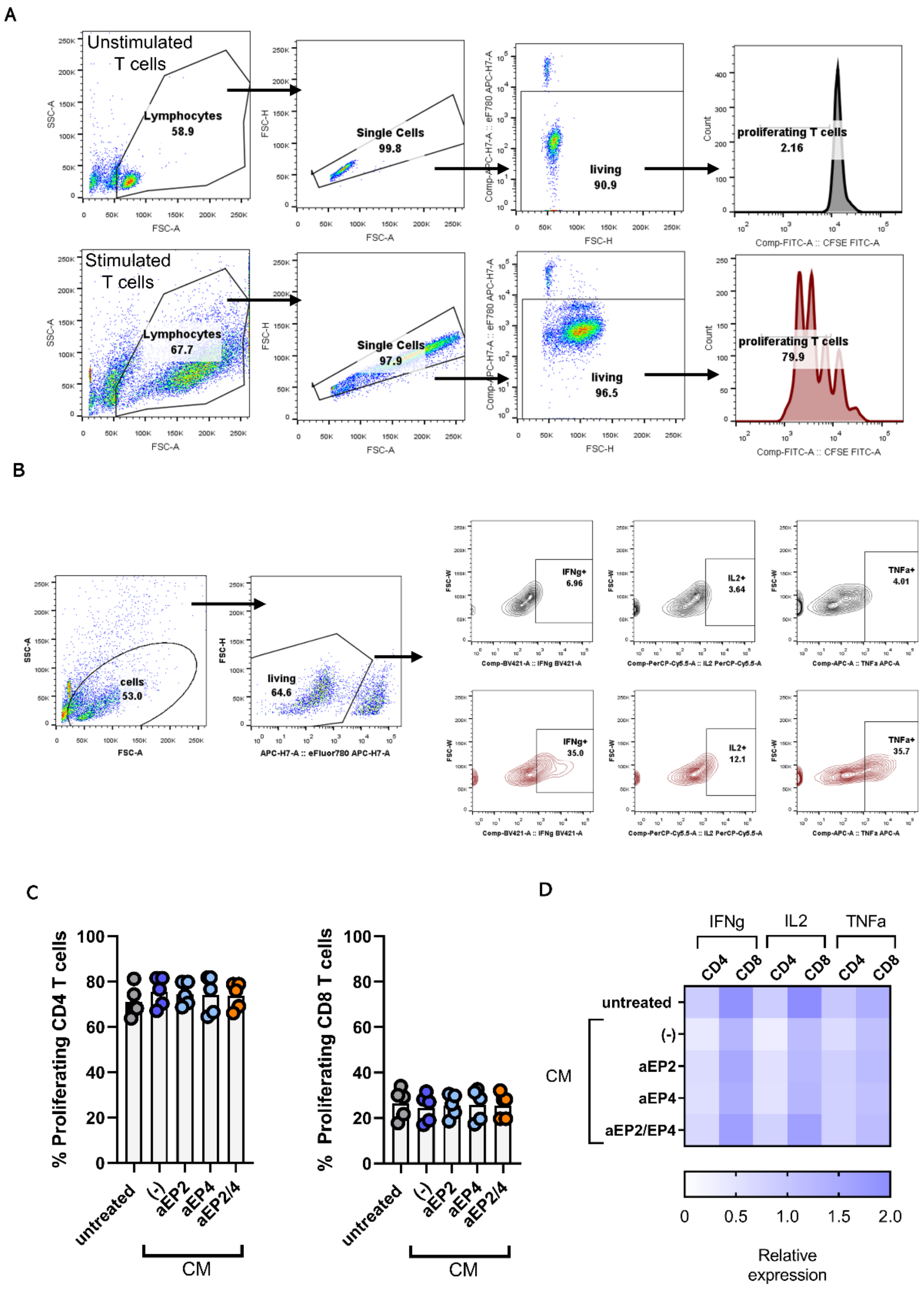

Supplementary Figure 3. Gating strategy to determine T cell phenotype and supplementary phenotype data. (A) Gating strategy to assess T cell proliferation. A first gate is set based on the physical parameters FCS-A and SSC-A, followed by a second gate on viable cells (eFluor780 negative population). CFSE dye on T cells enables the identification of the percentage of proliferating T cells by comparing with unstimulated T cells (non-proliferative). (B) Gating strategy to assess the intracellular cytokine production of autologous T cells. Expression of the indicated cytokine was

determined by comparing stained T cells (depicted in the red contour plots) with unstained T cells for the indicated fluorochrome (depicted as the grey contour plot). (C) Percentage of CD4 and CD8 T cells across the proliferating T cells from the autologous T cell proliferation assays. (D) Heatmap displaying the relative expression of IFNg, IL-2 and TNFa across CD4 and CD8 T cells from the autologous T cell cytokine suppression assays. Relative cytokine expression (ranging from 0-2) was calculated by normalizing to the expression levels of IFNg, TNFa and IL-2 given by untreated CD3 cells for every donor.

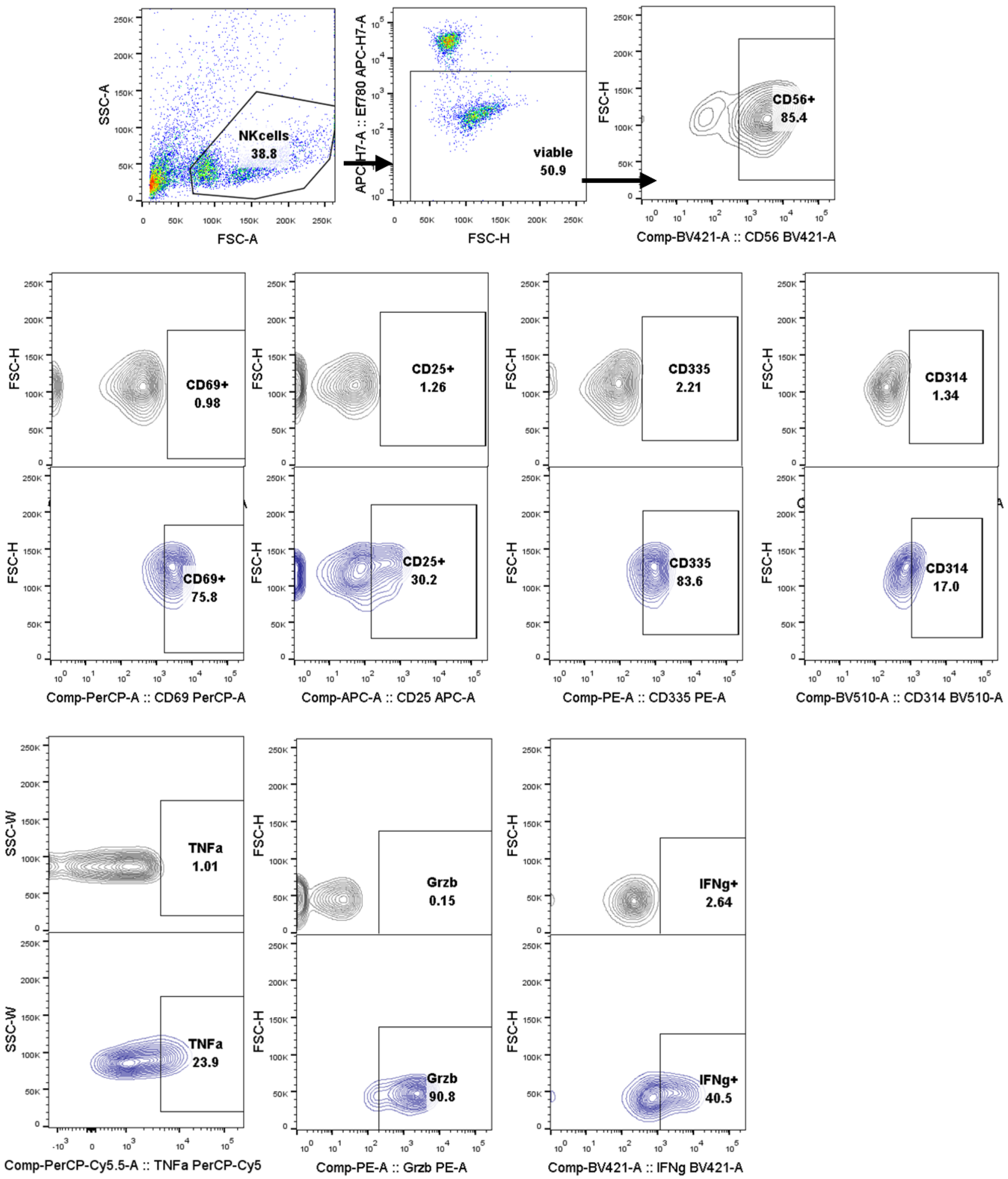

Supplementary Figure 4. Gating strategy to determine NK cell phenotype. A first gate is set based on the physical parameters FCS-A and SSC-A, followed by a second gate on viable cells (eFluor780 negative population). NK cells are then identified based on CD56 expression. Then, expression of the indicated surface marker or intracellular cytokine is determined by comparing stained NK cells (depicted in the blue contour plots) with unstained NK cells for the indicated fluorochrome (depicted as the grey contour plot).

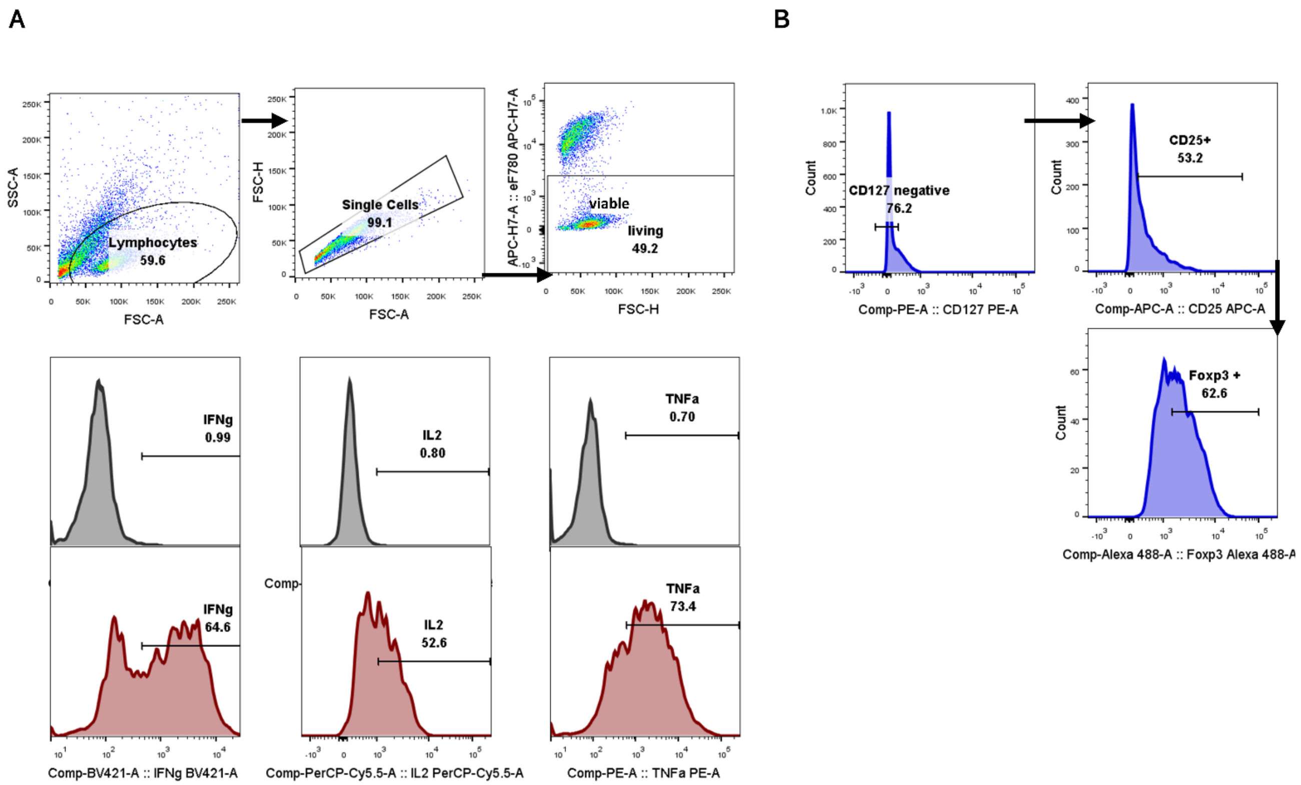

Supplementary Figure 5. Gating strategy to determine T cell phenotype. (A) Gating strategy to assess the intracellular cytokine production of polarized CD4 T cells. A first gate is set based on the physical parameters FCS-A and SSC-A, followed by a second gate on viable cells (eFluor780 negative population). Expression of the indicated cytokine was determined by comparing stained T cells (depicted in the red contour plots) with unstained T cells for the indicated fluorochrome (depicted as the grey contour plot). (B) Gating strategy for the identification of Tregs. After selecting on viable cells, Tregs are identified by negatively selecting for CD127 T cells (CD127 negative) followed by positively identifying the CD25 and FOXP3 positive T cells.

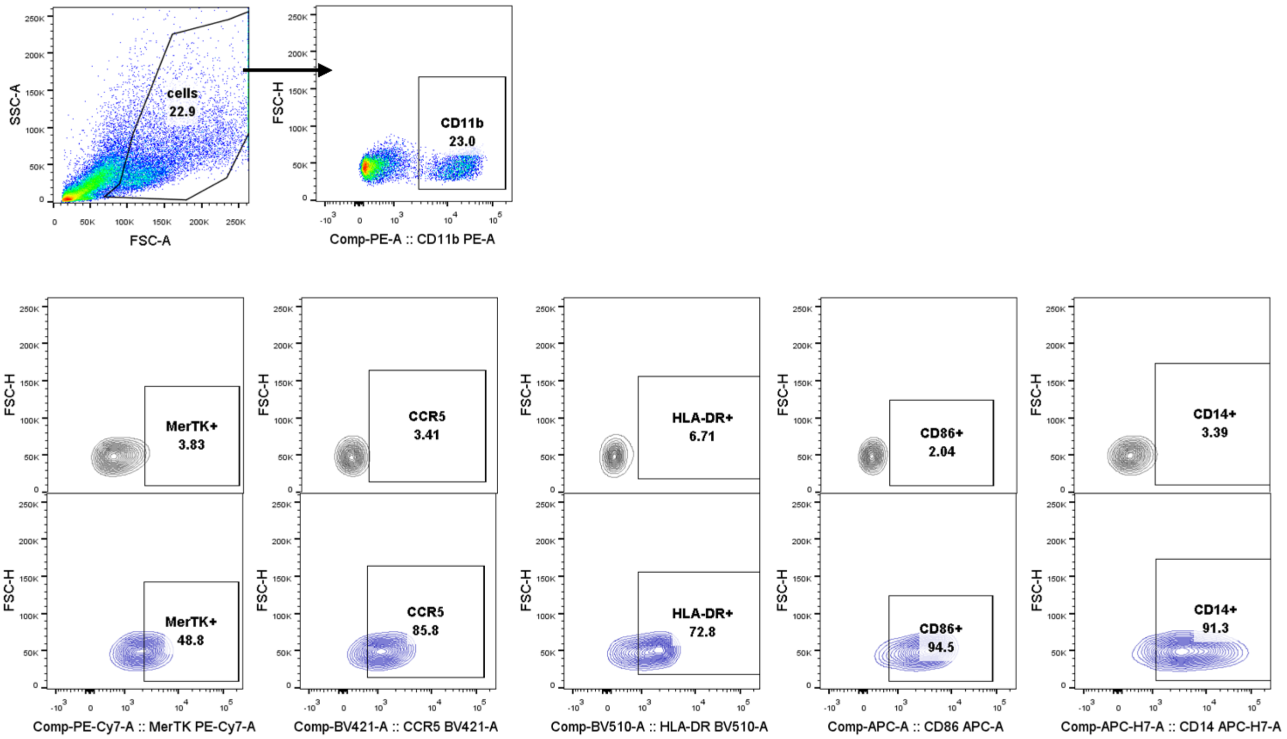

Supplementary figure 6. Gating strategy to assess the phenotype of moMDSCs in coculture with CRC PDTOs. An initial gate is set based on the physical parameters FCS-A and SSC-A. To identify MDSCs the positive expression levels of CD11b is used. Further phenotype characterization is then performed on CD11b cells. Expression of the indicated myeloid markers were determined by comparing stained samples (depicted as a blue contour plots) with unstained samples for the indicated fluorochrome (depicted as the grey contour plot).
